# DNA methylation landscape of cerebrospinal fluid cells in multiple sclerosis: an epigenome-wide association study

**DOI:** 10.1016/j.ebiom.2026.106454

**Published:** 2026-08-27

**Authors:** Yanan Han, Galina Yurevna Zheleznyakova, Heng Liang, Chiara Sorini, Majid Pahlevan Kakhki, Nicolas Ruffin, Nils Hallén, Chandana Rao Prakash, Viveca Beckers, Elena Ivanova, Mohsen Khademi, Tomas Olsson, Mikael C.I. Karlsson, Fredrik Piehl, Gavin Kelsey, Lara Kular, Maria Needhamsen, Maja Jagodic

**Affiliations:** a Department of Clinical Neuroscience, Karolinska Institutet, Center for Molecular Medicine, Karolinska University Hospital, Stockholm, Sweden; b Department of Microbiology, Tumor and Cell Biology, Karolinska Institutet, Stockholm, Sweden; c Epigenetics Programme, The Babraham Institute, Babraham Research Campus, Cambridge, UK; d Metabolic Research Laboratory, Wellcome-MRC Institute of Metabolic Science, Cambridge, UK; e Loke Centre for Trophoblast Research, Department of Physiology, Development and Neuroscience, University of Cambridge, Cambridge, UK

**Keywords:** Multiple sclerosis, DNA methylation, CSF immune cells, T cells, Protocadherins

## Abstract

**Background:**

Multiple sclerosis (MS) is a chronic inflammatory disease of the central nervous system in which DNA methylation may link genetic and environmental risk factors.

**Methods:**

We profiled genome-wide DNA methylation in cerebrospinal fluid (CSF) cells from people with MS (pwMS) and matched controls. Differentially methylated positions (DMPs) and regions (DMRs) were integrated with transcriptomic data, T-cell chromatin annotations, and pathway analyses. Protocadherin gamma (PCDHγ) expression was assessed in primary CD4+ T-cell subsets and confirmed by flow cytometry.

**Findings:**

We identified 2710 DMPs and 4330 DMRs associating with genes that were enriched in immune signalling, adhesion and migration processes, and were accompanied by corresponding RNA changes. MS-associated methylation changes enriched in the cohesin chromatin-regulation pathway localised to T-cell regulatory regions, and this pathway included multiple protocadherin (*PCDH*) genes, which displayed consistent methylation and expression changes in CSF cells of pwMS compared to controls. PCDHγ cluster gene expression was detected in CD4^+^ T-cell subsets, and flow cytometry confirmed PCDHγ protein expression in peripheral blood T cells. Moreover, co-expression analysis suggests a role of *PCDH* genes in aryl hydrocarbon receptor (AHR) signalling. Protein-level validation showed fewer PCDHγ-positive CD4+ T cells in pwMS and activation-induced PCDHγ upregulation after T-cell stimulation.

**Interpretation:**

DNA methylation changes in CSF resident cells reflect dysregulated T cell activation and migration in pwMS and suggest involvement of protocadherin molecules in MS pathogenesis.

**Funding:**

European Research Council, Swedish Research Council, Swedish Brain Foundation, Swedish MS Foundation, Knut and Alice Wallenberg Foundation, European Union and others.

**Research in context**

**Evidence before this study**

DNA methylation has been implicated in multiple sclerosis (MS), particularly at immune regulatory loci including the HLA region and *HLA-DRB1*. Previous epigenetic studies have largely examined peripheral blood or purified peripheral immune-cell subsets, identifying MS-associated methylation changes in CD4+ T cells, CD8+ T cells, B cells, and monocytes. In parallel, transcriptomic and single-cell studies of cerebrospinal fluid (CSF) have revealed compartmentalised immune activation, T-cell trafficking, clonal expansion, and inflammatory signalling in MS. However, peripheral blood may not fully capture CNS-proximal immune processes, and transcriptomic profiles provide limited information on stable or historical regulatory states. To the best of our knowledge as of July 2026, a genome-wide DNA methylation map of CSF-resident immune cells in MS, integrated with gene expression, chromatin-state annotations, and protein-level validation, has been lacking.

**Added value of this study**

This study provides a comprehensive genome-wide DNA methylation analysis of CSF cells from people with relapsing-remitting MS and neurological controls. We identified 2710 differentially methylated positions and 4330 differentially methylated regions, with reproducibility supported by technical and biological validation. These changes converged on immune signalling, cytokine responses, GPCR/Rho GTPase pathways, adhesion, and migration, and were accompanied by corresponding RNA expression changes. Integration with T-cell chromatin annotations showed that MS-associated methylation changes were enriched in regulatory elements, including Th17-cell enhancers. Unexpectedly, we identified coordinated methylation and expression changes affecting *protocadherin* *(PCDH)* genes, detected PCDHγ expression in CD4+ T-cell subsets, and validated altered PCDHγ protein expression in T cells from pwMS.

**Implications of all the available evidence**

Together, the available evidence indicates that CSF immune cells in MS carry disease-associated epigenetic programmes linked to T-cell activation, adhesion, and migration, potentially supporting immune-cell entry, persistence, or function within the CNS inflammatory compartment. The identification of protocadherin dysregulation in T cells extends the relevance of this gene family beyond neuronal adhesion and suggests a previously unrecognised role for PCDHγ in MS-associated immune regulation. Links between protocadherin-associated networks, cohesin/CTCF chromatin regulation, γ-secretase signalling, and AHR signalling nominate a regulatory axis through which genetic, environmental, metabolic, or viral cues may shape pathogenic immune responses. These findings support future single-cell epigenomic, longitudinal, and mechanistic studies to evaluate these signatures as biomarkers and therapeutic targets in MS.

## Introduction

Multiple sclerosis (MS) is a chronic autoimmune disease of the central nervous system (CNS) affecting approximately 2.8 million individuals worldwide.^1^^,^^2^ Initially, the disease is typically characterised by recurrent relapses driven by autoimmune-mediated inflammatory attacks on the CNS, followed by periods of remission, referred to as relapsing-remitting MS (RRMS).^3^ Although current immunomodulatory therapies can alleviate symptoms and drastically reduce relapse rates, none provide a cure and their impact on slowing down disease progression remains to be established.^4^ Individual's genetic background contributes to both the susceptibility and severity of MS^5^, ^6^, ^7^ while environmental exposures, including Epstein–Barr virus (EBV) infection, vitamin D deficiency, and smoking, further modulate disease risk and progression.^8^ Recent compelling evidence suggests that genetic risk variants and EBV infection act synergistically to disrupt immune tolerance, thereby promoting autoreactive T cell responses against CNS antigens.^9^, ^10^, ^11^, ^12^, ^13^ Despite these insights, molecular mechanisms linking the risk factors to pathogenic immune activity remain incompletely understood, limiting our ability to develop more precise therapies.

This gap of knowledge has led to increasing interest in epigenetic mechanisms, which may mediate the interaction between genes and environmental exposures and serve as a molecular interface between the risk factors and altered immune cell functions in people with MS (pwMS).^14^ DNA methylation, the most extensively studied epigenetic modification, involves the addition of a methyl group to cytosine residues, typically at CpG dinucleotides. When present at promoter or enhancer regions, DNA methylation can inhibit transcription by blocking binding of transcription factors or by recruiting repressive chromatin complexes. DNA methylation has been reported to mediate the effects of genetic and lifestyle factors in MS.^14^, ^15^, ^16^, ^17^ Moreover, large-scale and multi-cell-type studies consistently identified the human leucocyte antigen (HLA) locus, in particular the strongest MS risk gene, *HLA-DRB1∗15:01*, as the most robust region of differential methylation in pwMS.^14^^,^^15^^,^^18^^,^^19^ The *HLA-DR* genes encode the major histocompatibility complex (MHC) class II molecules that present antigens to CD4^+^ T cells, strongly implicating the critical role of CD4^+^ T helper (Th) cells in the breakdown of tolerance to self-antigens in pwMS. *HLA-DRB1∗15:01* is associated with allele-specific DNA hypomethylation and increased expression in immune cells, linking genotype to altered epigenetic regulation and immune activation.^14^^,^^15^ Beyond HLA, DNA methylation also regulates genes encoding lineage-defining transcription factors, such as retinoic acid receptor-related orphan receptor gamma t (*RORC*) and forkhead box P3 (*FOXP3*), shaping the balance between pro-inflammatory Th17 and regulatory (Treg) cells.^20^, ^21^, ^22^, ^23^ Disruption of this balance is strongly implicated in MS pathogenesis.^24^, ^25^, ^26^ However, the vast majority of methylation studies have focused on peripheral blood, where pathogenic T cell subsets are rare and may not fully reflect compartmentalised CNS responses.

Cells circulating in the cerebrospinal fluid (CSF) provide a unique window into the immune processes occurring within the CNS, as CSF resides in close proximity to demyelinating lesions and reflects active neuroinflammation. CSF cells are predominantly composed of T lymphocytes, with CD4^+^ T cells representing the major population. Comparative profiling of CSF cells and peripheral blood mononuclear cells (PBMC) has shown that CSF cells exhibit stronger pathogenic signatures in MS, with CD4^+^ T cells displaying heightened expression signatures of activation, tissue adaptation, and trafficking in CSF.^27^, ^28^, ^29^ Recent single-cell studies have further highlighted MS-specific immune states within the CSF, revealing broad cellular and transcriptional alterations in neuroinflammation,^30^ the presence of CNS-resident immune cells detectable in CSF,^31^ T follicular helper (Tfh) cells that can facilitate B cell infiltration into the CNS,^32^ and clonally expanded cytotoxic T cell populations in pwMS.^33^^,^^34^ These cellular states are marked by transcriptional programmes characterised by T cell activation, interferon (IFN) and tumour necrosis factor (TNF) signalling and cytotoxicity.^33^ Although single-cell transcriptomics has provided important insights, its limited gene coverage hinders the detection of lowly expressed functionally critical regulators such as transcription factors. Moreover, transcriptomic profiles offer only a cross-sectional snapshot of cellular states at the time of sampling, in contrast to epigenetic modifications that can capture more stable or historical regulatory imprints.

In this study, we sought to elucidate the molecular mechanisms underlying MS immunopathology by identifying and functionally characterising genome-wide DNA methylation changes in CSF cells, which are enriched for pathogenic immune activity. A sequencing approach optimised for low-input samples^35^^,^^36^ enabled us to generate a comprehensive methylome of CSF infiltrating cells. We integrated T cell subset-specific regulatory annotations from the International Human Epigenome Consortium (IHEC, preprint, not peer-reviewed)^37^ to infer cellular functions and pathways affected by genomic regions displaying MS-associated differential methylation. Furthermore, to pinpoint expression changes of the identified MS-associated molecules across immune cell subtypes, we leveraged locally generated T cell subset datasets. Together, these approaches provide an integrated view of the CSF immune-cell epigenome and reveal MS-associated regulatory Programmes governing altered immune cell adhesion, migration, and function.

## Methods

### Cohorts

CSF cells from 5 RRMS and 5 NINDC were included in the genome-wide post-bisulfite adaptor tagging (PBAT) discovery cohort. Technical validation using a less deep WGBS PBAT sequencing was performed on the same samples, as well as on an additional independent cohort 18 RRMS and 7 NINDC and 5 INDC. Of those, 11 RRMS and 6 NINDC CSF cell samples were profiled with RNA sequencing (RNA-seq).^29^ All pwMS were diagnosed according to 2010 revised McDonald's criteria. Patients’ Expanded Disability Status Scale (EDSS) and the Multiple Sclerosis Severity Score (MSSS) were determined by the treating neurologist. Detailed demographic and clinical characteristics of the study participants are provided in Supplementary Tables S1–S3. Treatment status was not included as a covariate in the statistical models because only a small number of participants were receiving treatment at the time of sampling and the treatment categories were heterogeneous, precluding reliable estimation of treatment-specific effects. The study was approved by the regional ethical committee (ethical permit 2009/2107-31/2) and written informed consent was obtained from all participants.

All eligible CSF samples were initially sequenced. For the high-depth discovery cohort, we aimed to select a balanced set of RRMS and NINDC samples to reduce disease-group imbalance, while prioritising libraries with sufficient complexity and post-deduplication CpG coverage. The 10 samples included in the discovery cohort were also profiled by lower-depth WGBS PBAT sequencing to assess technical reproducibility. In addition, an independent validation cohort comprising 30 non-overlapping donors was profiled by lower-depth WGBS PBAT sequencing for validation of selected candidate findings. Assignment to deeper sequencing was based on library complexity and post-deduplication CpG coverage and was independent of the observed methylation results. Samples with sufficiently complex libraries benefited from deeper sequencing and were included in the discovery cohort. Samples with low-complexity libraries, for which deeper sequencing predominantly generated duplicate reads and provided limited improvement in CpG coverage after deduplication, were retained for lower-depth validation rather than discovery-level sequencing.

### Preparation of CSF cells

The CSF was collected in 2 × 15 ml size falcon tubes and centrifuged immediately after lumbar puncture at 440 g for 10 min at RT to separate the cells, which were subsequently pooled and concentrated to a volume of 20–60 μl per sample. The CSF cells were then frozen immediately on dry-ice and stored in 2 ml size polypropylene tube at −80 °C until use.

### Library preparation

DNA methylation libraries were prepared using the Post-Bisulfite Adaptor Tagging (PBAT) method^35^ with some modifications. Lysates from ∼5000 CSF cells were bisulfite converted using the Imprint DNA Modification Kit (Sigma) by incubation at 99 °C for 6 min, 65 °C for 80 min, 95 °C for 3 min and 65 °C for 20 min. Purification was done according to the manufacturer's protocol. DNA was eluted in 10 mM Tris-Cl (pH 8.5) and mixed with 0.4 mM dNTPs, 0.4 uM oligo 1 (Biotin)CTACACGACGCTCTTCCGATCTNNNNNNNNN), 1× NEBuffer 2 with a final reaction volume of 24 μl. The samples were incubated at 65 °C for 3 min followed by 4 °C pause, 5 U of Klenow exo- (New England Biolabs) was added and the incubation was continued at 4 °C for 5 min, +1 °C/15 s to 37 °C, 37 °C for 90 min. Samples were then incubated with 20 U exonuclease I (NEB) for 1 h at 37 °C. DNA was purified with 0.8× Agencourt Ampure XP beads (Beckman Coulter), eluted in 10 mM Tris-Cl (pH 8.5) and incubated with washed M−280 Streptavidin Dynabeads (Invitrogen) for 30 min with rotation at room temperature. Beads were washed twice with 0.1 N NaOH, and twice with 10 mM Tris-Cl (pH 8.5) and resuspended in 47 μl of 0.4 mM dNTPs, 0.4 μM oligo 2 (TGCTGAACCGCTCTTCCGATCTNNNNNNNNN) and 1× NEBuffer 2. Samples were then incubated at 95 °C for 45 s followed by 4 °C pause before addition of 10 U Klenow exo- and then incubated at +1 °C/15 s to 37 °C, 37 °C for 90 min. Beads were washed with 10 mM Tris-Cl (pH 8.5) and resuspended in 50 μl of 0.4 mM dNTPs, 0.4 μM PE1.0 forward primer (AATGATACGGCGACCACCGAGATCTACACTCTTTCCCTACACGACGCTCTTCCGATCT), 0.4 μM indexed iPCRTag reverse primer, 1 U KAPA HiFi HotStart DNA polymerase (KAPA Biosystems) in 1× HiFi Fidelity Buffer. Libraries were amplified by PCR as follows: 95 °C 2 min, 12–15 cycles of (98 °C 80 s, 65 °C 30 s, 72 °C 30 s), 72 °C 3 min and 4 °C hold. Amplified libraries were purified using 0.8× Agencourt Ampure XP beads. Libraries' quality and quantity were determined using High-Sensitivity DNA chips on the Agilent Bioanalyzer, and the KAPA Library Quantification Kit for Illumina (KAPA Biosystems). Pools of libraries were prepared for HiSeq 100 bp Single-end sequencing on a HiSeq2500 in High Output mode (8 lanes/run) at the Babraham Institute Next Generation Sequencing Facility. 5 RRMS and 5 NINDC samples were sequenced with 150 bp paired end reads on 20 HiSeqX lanes (2 lanes per sample) at the National Genomics Infrastructure (NGI, Stockholm).

### Trimming and filtering of sequencing reads

Single end reads of 100 bp were processed using Trim Galore! (v0.4.1) developed by Babraham Bioinformatics, with the first 9 bp clipped from the 5′ end (--clip_R1 9). Quality (-q 20) and adaptor (-a AGATCGGAAGAGC) trimming were performed using Cutadapt (v1.8.1)^38^ with a minimum required adaptor overlap of 1bp (-O 1) and a maximum trimming error rate of 0.1 (-e 0.1). Reads shorter than 20 bp after trimming were discarded. Trimmed and filtered sequencing reads were aligned to GRCh38 with the -pbat^35^ option of Bismark (v0.14.4),^39^ using Bowtie 2 and the following specified options: -q -phred33 -score-min L,0,-0.2 -ignore-quals. Alignments with a unique best hit were taken for further processing.

### Statistics

#### Estimation of DNA methylation

Duplicated reads were removed using the deduplicate_bismark function and subsequently top and bottom strands were merged into single CpG dinucleotides using the -merge_CpG option of the Bismark coverage2cytosine module.^39^ Methylation level values were calculated by reads mapped as cytosine divided by total coverage. CpG with coverage ≥10 in more than 8 samples were kept for downstream analysis and methylation level were NA if the coverage is less than 10. Chromosome X and Y were removed. Gene and genomic feature annotations were obtained from Ensembl,^40^ GRCh38, gene were extended upstream for 2 kb. BEDTools^41^ were used to intersect the CpGs. Methylation pattern analyses were performed by CpGtools.^42^

#### Cell-type proportion estimation

Immune cell-type composition in cerebrospinal fluid (CSF) samples was estimated by reference-based deconvolution of DNA methylation data using EpiDISH (v2.22.0; CBS method).^43^ We used the cent12CT.m reference matrix comprising 12 immune cell subtypes.^44^ Reference CpG probes were lifted from hg19 to hg38 coordinates and matched to CpG sites covered in each sample. Methylation beta values overlapping reference CpGs were retained for deconvolution, resulting in variable CpG overlap across donors (112–513 CpGs). Cell-type fractions were estimated using the epidish function and reported as proportions of the 12 immune cell subtypes. Estimated proportions between RRMS and NINDC were compared using two-sided Wilcoxon rank-sum tests. *P* values from the cell-type comparisons were adjusted for multiple testing across cell types using the Benjamini–Hochberg method. Standardised effect sizes were calculated as Cohen's d using the pooled within-group standard deviation. Cell-type proportions are visualised as boxplots showing medians, interquartile ranges, and individual samples.

#### Identification of DMPs

We used three established methods representing complementary statistical frameworks to identify candidate DMPs. Limma regression, which uses empirical Bayes moderation, allowing us to consider covariates such as age and sex.^45^ MethylKit utilises logistic regression,^46^ while RADmeth takes advantage of Beta-binomial distribution.^47^ Because no single method is universally considered optimal for differential methylation analysis, and because methods based on different statistical assumptions may produce different results depending on the characteristics of the data, we adopted a consensus-based approach. The two-of-three criterion was adopted as a pragmatic robustness filter to prioritise CpG sites supported by more than one analytical framework and reduce reliance on any single model, rather than as a formal method for combining statistical evidence.

For Limma and RADmeth, the previously mentioned filtering strategy, which retained CpG sites with a minimum coverage of 10× in at least eight samples, was used. In the case of methylKit, a uniform minimum coverage threshold of 10× was applied across all samples, as the method requires consistent coverage. Sex and age were included as covariates in both Limma and methylKit models. CpG sites which met the criteria of *P* < 0.001 and absolute methylation differences ≥0.1 in at least two of the three methods were defined as DMPs. Because approximately 12 million CpG sites were assessed in the discovery cohort, conventional genome-wide multiple-testing correction yielded no significant CpGs. Candidate DMPs were therefore selected using nominal, unadjusted *P* values together with an absolute methylation-difference threshold of ≥10% and concordant support from at least two of the three complementary analytical methods. This consensus-based strategy was intended to reduce reliance on any single statistical framework and prioritise robust candidates, although it does not provide formal genome-wide false-discovery-rate control.

#### Identification of DMRs

To identify DMRs, CpG sites with a *P* < 0.01 and an absolute methylation difference ≥0.1 determined by any of the three methods, were selected as candidate sites for DMR construction. Candidate CpGs were then clustered by BEDTools with a maximum inter-CpG distance of 1000 bp, applying a strand-specific mode (the direction of methylation level change). Regions containing more than three candidate CpG sites were retained and defined as DMRs. These predefined criteria were used to prioritise spatially coherent methylation changes supported by multiple neighbouring CpGs rather than isolated CpG-level signals. The 1000 bp distance threshold and the requirement for multiple CpGs per region are consistent with commonly used DMR detection frameworks, including dmrseq^48^ and DMRcate,^49^ which use similar distance-based aggregation and minimum CpG-support criteria for candidate regions. The *P* values used to select candidate CpGs for DMR construction were nominal and unadjusted. Robustness was instead supported by the ≥10% methylation-difference threshold, concordant direction of methylation change, spatial proximity, and the requirement for more than three CpGs per region. This approach does not provide formal genome-wide false-discovery-rate control.

#### Enrichment analysis of DMRs near MS risk loci

MS risk loci were defined based on lead SNPs reported by the International Multiple Sclerosis Genetics Consortium.^5^ Genomic coordinates were harmonised to the hg38 reference genome using UCSC liftOver. Each SNP was extended to a 4 kb window centred on the lead variant (±2 kb). Enrichment of DMRs overlapping these regions was assessed using a permutation-based approach. DMR coordinates were randomly shifted 5000 times across the genome while preserving region length, and the number of overlaps was calculated for each iteration. An empirical *P* value was derived as the proportion of permutations yielding overlap counts greater than or equal to the observed value.

#### Replication cohort data analysis

To improve precision and enable the use of CpG sites with lower coverage, weighted local regression (BSmooth)^50^ was applied with default parameters to smooth methylation profiles, with weights determined by read coverage. Smoothed methylation levels were used as input to estimate differential methylation between RRMS and INDC and NINDC control samples. CpG sites exhibiting consistent directional changes across both deep and shallow sequencing datasets, for the 10 technical replicate samples, were used as input for the independent cohort comparison, where quadrant plots were generated.

#### DMP and DMR's annotation

Gene and genomic feature annotations were obtained from Ensembl,^40^ based on the GRCh38 reference genome. Gene coordinates were extended 2 kb upstream of the transcriptional start site to include promoters. CpG sites were intersected with genomic features using BEDtools.^41^ CpG island annotations were downloaded from UCSC Genome Browser hg38.^51^ DNA methylation patterns across genomic features were explored using CpGtools.^42^ EpiLogos was used to visualise epigenomic feature around DMRs overlapping MS risk SNPs.^52^

#### Function analysis and regulator prediction

Genes associated with DMPs and DMRs were subjected to Ingenuity Pathway Analysis (IPA, QIAGEN) for functional annotation and upstream regulator prediction.^53^ Enrichment significance was determined using Benjamini-Hochberg (BH)-adjusted *P* values. Significantly enriched pathways (Padj <0.05 and molecule count ≥10) were grouped using GeneSetCluster (version 2.0).^54^ The optimal number of pathway clusters was evaluated using the OptimalGeneSets function implemented in GeneSetCluster 2.0. Hierarchical clustering solutions containing 2–15 clusters were assessed using average silhouette width (method = “silhouette”, cluster_method = “hcut”, and max_cluster = 15), with the solution yielding the highest average silhouette width selected as optimal. IPA was also applied to explore subsets of genes with 1) differential promoter methylation and inverse expression changes, 2) DMP and DMR associated genes within different IHEC (preprint, not peer-reviewed)^37^ features and cell types, 3) genes within PCDH clusters, and 4) target genes of generic transcriptional regulatory pathways. Genomic tracks were visualised using the Integrative Genomics Viewer.^55^

#### IHEC enrichment and functions

Individual chromatin states from 229 T cell samples (healthy donors), as well as summary chromHMM annotations were extracted from IHEC (preprint, not peer-reviewed).^37^ Enrichment analysis was performed using the ChromHMM OverlapEnrichment (-center) tool^56^ with default parameters. UpsetR^57^ was used to visualise overlapping genes across different T cell types and genomic features. We next conducted IPA analysis on the differentially methylated genes mapping to four chromatin state groups TSS, Enh, EnhBiv and ReprPC in a given T cell subset. The final example of RAC2 was shown in integrative genomics viewer (IGV).^55^

#### CD4^+^ T cell subset dataset

The CD4+ T cell subsets were isolated from peripheral blood of six healthy donors using fluorescence-activated cell sorting (Supplementary Table S11). RNA was extracted directly following sorting using AllPrep DNA/RNA Mini Kit (Qiagen) and RNA-seq libraries were constructed using the Illumina® Stranded Total RNA Prep with Ribo-Zero Plus (Illumina, CA, USA) according to the manufacturer's protocol. Libraries were sequenced on an Illumina NovaSeq 6000 instrument using 150 nt paired end reads. The Bcl to FASTQ conversion was performed using bcl2fastq from the CASAVA software suite at NGI, Stockholm, Sweden. Raw reads were processed using the nf-core/rnaseq pipeline (version 3.3),^58^ reads were adaptor-trimmed and quality-filtered using cutadapt (v2.10) and filtered reads were aligned to the human genome (GRCh38) using STAR (v2.7.6a)^59^ with default parameters. Read counts were imported into R using the tximport package (v. 1.18.0)^60^ with the tx2gene and lengthScaledTPM parameters.

#### Flow cytometric staining of pan-PCDHγ

Human peripheral blood mononuclear cells (PBMCs) were stained using a pan-PCDHγ antibody panel and analysed on a BD LSRFortessa flow cytometer. Briefly, 1 × 10^6^ cells were incubated with a fixable viability dye and Fc receptor block. Cells were then fixed with 3.65% formaldehyde for 20 min and permeabilised with 0.1% IGEPAL CA-630 for 4 min to enable intracellular detection of pan-PCDHγ. A mouse anti-pan-PCDHγ antibody (Abcam, cat.no. ab187186), was used to detect the C-terminus region common to multiple members of the PCDHγ family, and signal was amplified by staining with a secondary AF488-conjugated anti-mouse IgG antibody (Abcam, cat.no. ab150113). Subsequently, staining of lineage-defining surface markers was carried out using antibodies against CD3 (BioLegend, cat.no. 300328), CD20 (Miltenyi, cat.no. 130-111-338).

To analyse PCDHγ expression in T cell subsets, PBMCs from 3 healthy donors and 3 pwMS were stained with an expanded antibody panel including CD3, CD4 (BD, cat.no. 557852) and CD45RO (BioLegend, cat.no. 304251) to assess pan-PCDHγ expression in naïve-like (CD45RO^−^) and memory (CD45RO^+^) CD4^+^ T cells. Cells were gated sequentially on lymphocytes, single cells, live cells, CD3^+^ cells and CD4^+^ cells, followed by separation into CD45RO^−^ and CD45RO^+^ subsets. The frequency of pan-PCDHγ^+^ cells was quantified within each CD4^+^ T cell subset.

For in vitro T cell stimulation, PBMCs were thawed, washed and cultured in R10 medium consisting of RPMI-1640 supplemented with 10% FBS, 1% l-glutamine and 1% penicillin-streptomycin. Cells were seeded at 2.5 × 10ˆ5 cells per well in 96-well U-bottom tissue culture plates and cultured for 24 h at 37 °C with 5% CO2. Cells were left unstimulated or stimulated with anti-CD3 mAb (Mabtech, cat# CD3-2) at a final 1:1000 dilution for 24 h. During the final 6 h prior to harvest, PMA/ionomycin Cell Stimulation Cocktail (1: 500) was added, and Brefeldin A solution (BioLegend, Cat# 420601) was included throughout this 6-h period to block protein secretion. After stimulation, cells were stained for CD69 (BioLegend, cat.no. 310906), TNFα (BioLegend, cat.no. 502920) and pan-PCDHγ. Data were analysed using FlowJo v10.8.1.

#### PCDH co-expression network

Transcript per million (TPM) values from RNA-seq data of naïve conventional CD4^+^ T cells (Tnv), T stem cell memory T cells (Tscm), T helper 2 cells (Th2), T helper 1 cells (Th1), T helper 17 cells (Th17), T helper 1/17 cells (Th1_17), circulating T follicular helper cells (cTfh), naïve regulatory T cells (Treg_nv), memory regulatory T cells (Treg_mem), terminally differentiated effector memory T cells re-expressing CD45RA (TEMRA) were used to generate the network. Genes with a summed TPM ≥1 across all samples were retained for correlation analysis. Pairwise gene correlations were calculated using Spearman's rank correlation coefficient. Gene pairs with an absolute correlation coefficient ≥0.5 and a Padj <0.01 were used to construct the co-expression network. Network Visualisation and construction were performed using Cytoscape.^61^ For descriptive interpretation, the PCDH co-expression network organised into three functional modules based on connectivity to distinct *PCDH* genes, expression patterns across T-cell subsets, and IPA functional enrichment results. These modules were used to summarise functionally related network components and were not derived by statistical optimisation of a predefined number of modules. Transcription factor–target regulons were obtained from the DoRothEA human database (dorothea_hs), using interactions with confidence levels A–C.^62^

Robust Multi-array Average (RMA) normalisation was applied to Affymetrix CSF microarray data to generate expression values for supplementary network construction.^28^^,^^63^ Genes with a summed expression value ≥ 10 were retained for downstream analysis. Pairwise gene correlations were calculated using Pearson correlation, and associations with the Padj <0.001 were used for IPA.

### Ethics

The CSF samples used for PBAT sequencing were obtained from the STOP-MS study, approved by the Regional Ethical Review Board in Stockholm (Regionala etikprövningsnämnden i Stockholm) under the original ethical permit no. 2009/2107-31/2, and subsequently amended by the Swedish Ethical Review Authority (most recent amendment 2025-04961-02). Written informed consent was obtained from all participants. Peripheral blood samples used for flow cytometry were obtained from the ResolveMS study, approved by the Swedish Ethical Review Authority (Etikprövningsmyndigheten) under the ethical permit no. 2020-06295. All procedures complied with the relevant ethical approvals and regulations.

### Role of funders

The funders had no role in study design, data collection, data analysis, data interpretation, writing of the manuscript, or the decision to submit the manuscript for publication. All authors had access to the data relevant to their contributions. The corresponding author had final responsibility for the decision to submit for publication.

## Results

### CSF cells display typical DNA methylation landscape

**Fig. 1:**
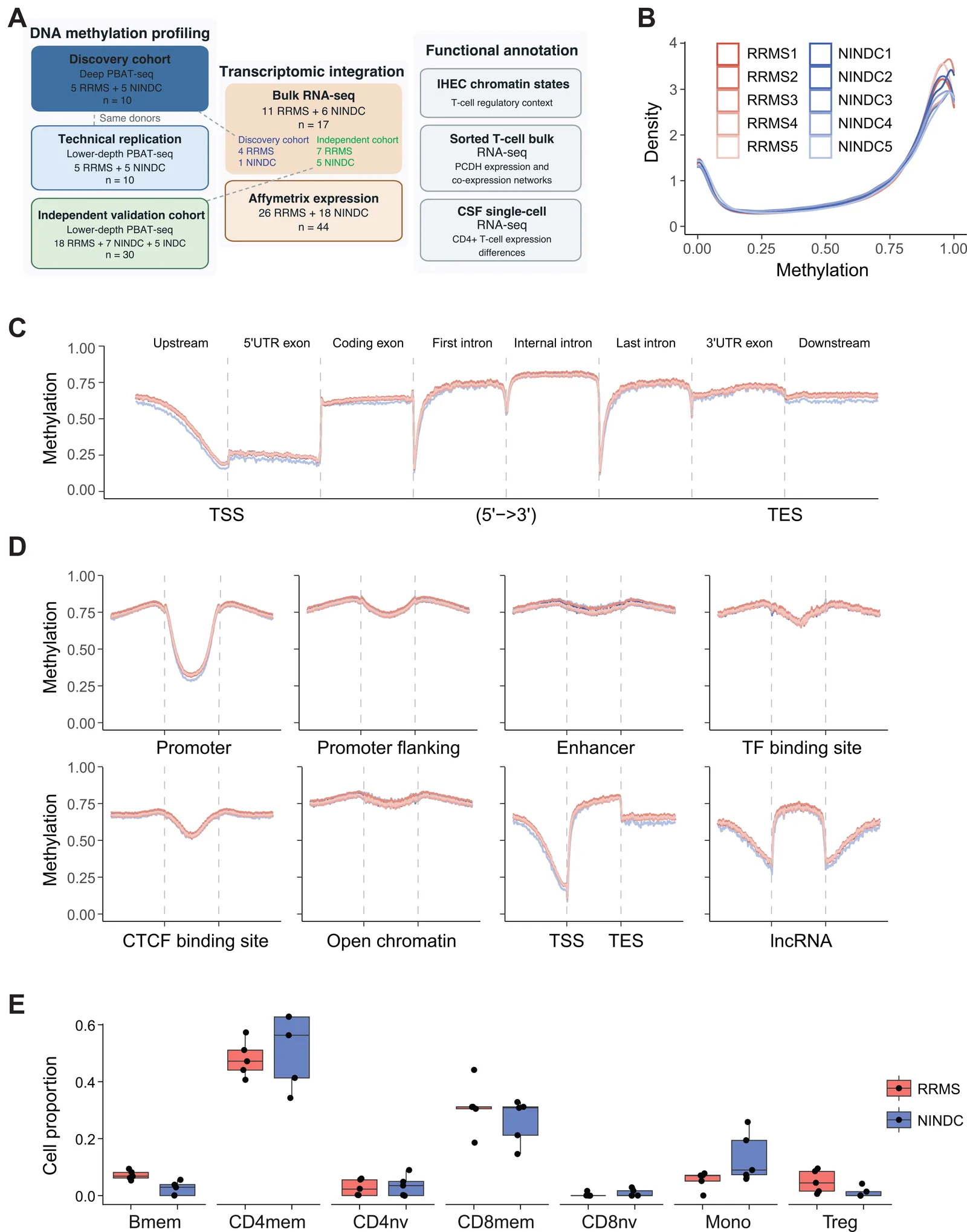
**DNA methylation profiling of CSF cells from RRMS and NINDCs**. (**A)** Schematic overview of the study design showing CSF cell collection from RRMS and NINDC donors followed by PBAT whole-genome bisulfite sequencing. **(B)** DNA methylation distribution across RRMS (n = 5) and NINDC (n = 5) samples **(C)** DNA methylation patterns across gene-related features. **(D)** DNA methylation patterns across Ensembl-derived genomic features. The dashed lines indicate the start and end of features. **(E)** Estimated proportions of immune cell subsets in CSF samples from individuals with RRMS and NINDC. Boxes represent the interquartile range, centre lines indicate medians, and whiskers denote 1.5× the interquartile range. Points represent individual samples. CSF: cerebrospinal fluid, RRMS: Relapsing–remitting multiple sclerosis, NINDC: non-inflammatory neurological disease controls, NDC: neurological disease controls, UTR: untranslated region, TSS: transcriptional start site and TES: transcriptional end site, CTCF: CCCTC-binding factor, lncRNA: long non-coding RNA, Treg: regulatory T cells, CD4mem: CD4^+^ memory T cells, CD4nv: CD4^+^ naïve T cells, CD8mem: CD8^+^ memory T cells, CD8nv: CD8^+^ naïve T cells, Bmem: memory B cells, Mono: monocytes.

To investigate genome-wide DNA methylation differences between RRMS and controls, we included two non-overlapping CSF donor cohorts. The discovery cohort comprised 10 donors (5 RRMS and 5 NINDC), for which PBAT libraries were generated and sequenced at high depth. The same 10 libraries were also sequenced at lower depth and served as technical replicates for evaluating concordance between sequencing depths. An independent validation cohort comprised 30 additional donors (18 RRMS, 7 NINDC, and 5 inflammatory neurological disease controls [INDC]), for which PBAT libraries were generated and sequenced at lower depth. Thus, the complete lower-depth sequencing dataset included 40 samples, of which 10 overlapped with the high-depth discovery cohort and 30 represented independent biological samples (Fig. 1A; Supplementary Tables S1 and S2).

To investigate the functional relevance of the methylation findings, we integrated two CSF transcriptomic datasets: RNA-seq data^29^ from 17 donors (11 RRMS and 6 NINDC), including donors overlapping with the methylation cohorts, and Affymetrix expression data^28^ from an independent cohort of 26 RRMS and 18 NINDC donors. We additionally used IHEC chromatin-state annotations of T cells^52^ to assess cell-type-specific regulatory contexts, bulk RNA-seq data from sorted T-cell subsets to characterise PCDH expression and construct gene co-expression networks, and single-cell RNA-seq data from CSF cells to examine gene-expression differences in CD4^+^ T cells (Fig. 1A; Supplementary Table S3).

In the discovery cohort, approximately 28 million CpG sites were sequenced genome-wide (Supplementary Fig. S1A and B), and 11.7 million sites remained for analysis after applying a coverage threshold of ≥10× in at least 8 of the 10 samples (Supplementary Fig. S1C). Density plots confirmed a typical bimodal distribution of methylation with the largest fraction of CpGs being highly methylated (>75% methylation) and comparably smaller number of CpGs being fully unmethylated (Fig. 1B). Low DNA methylation levels (<25%) were observed within and just upstream of 5’ untranslated regions (UTRs), whereas downstream genic regions displayed high (around 75%) DNA methylation levels (Fig. 1C). As expected, promoters and transcription start sites (TSSs) showed low methylation, followed by CTCF and transcription factors (TF) binding sites and promoter-flanking regions (Fig. 1D). A moderate dip in methylation was also observed in enhancers and open chromatin regions (Fig. 1D). Notably, DNA methylation landscape across lncRNA genes resembled that of protein-coding genes (Fig. 1D).

Cell-type deconvolution of CSF DNA methylation profiles revealed that T cells constituted the dominant immune population across all samples (Fig. 1E). Memory CD4^+^ T cells (CD4Tmem) represented the largest fraction in both groups, followed by CD8^+^ memory T cells. Comparative analysis between RRMS and NINDC samples demonstrated largely comparable overall immune cell compositions. No statistically significant differences were observed for Tregs, CD4Tmem, CD4Tnv, CD8Tmem, or CD8Tnv cells, with mean RRMS–NINDC differences of 3.79%, −3.46%, −0.69%, 4.98%, and −0.59% and corresponding standardised effect sizes (Cohen's d) of 1.22, −0.34, −0.21, 0.59, and −0.56, respectively. Monocytes showed a mean RRMS–NINDC difference of −8.08% (Cohen's d = −1.23; *P* = 0.095, BH-adjusted *P* = 0.221), whereas memory B cells showed a nominally significant mean RRMS–NINDC difference of 4.67% (Cohen's d = 2.27; *P* = 0.021), although this difference did not remain significant after multiple-testing correction (BH-adjusted *P* = 0.148). Overall, these findings indicate that CSF immune cell composition was broadly comparable between groups, with only modest shifts observed in specific B cell subsets.

Taken together, these results confirm the expected DNA methylation architecture of CSF cells across genomic and regulatory elements, suggest largely comparable immune cell compositions between groups, and support the suitability of PBAT for whole-genome methylome profiling in low-input clinical samples.

### Widespread DNA methylation alterations characterise CSF cells in pwMS

**Fig. 2:**
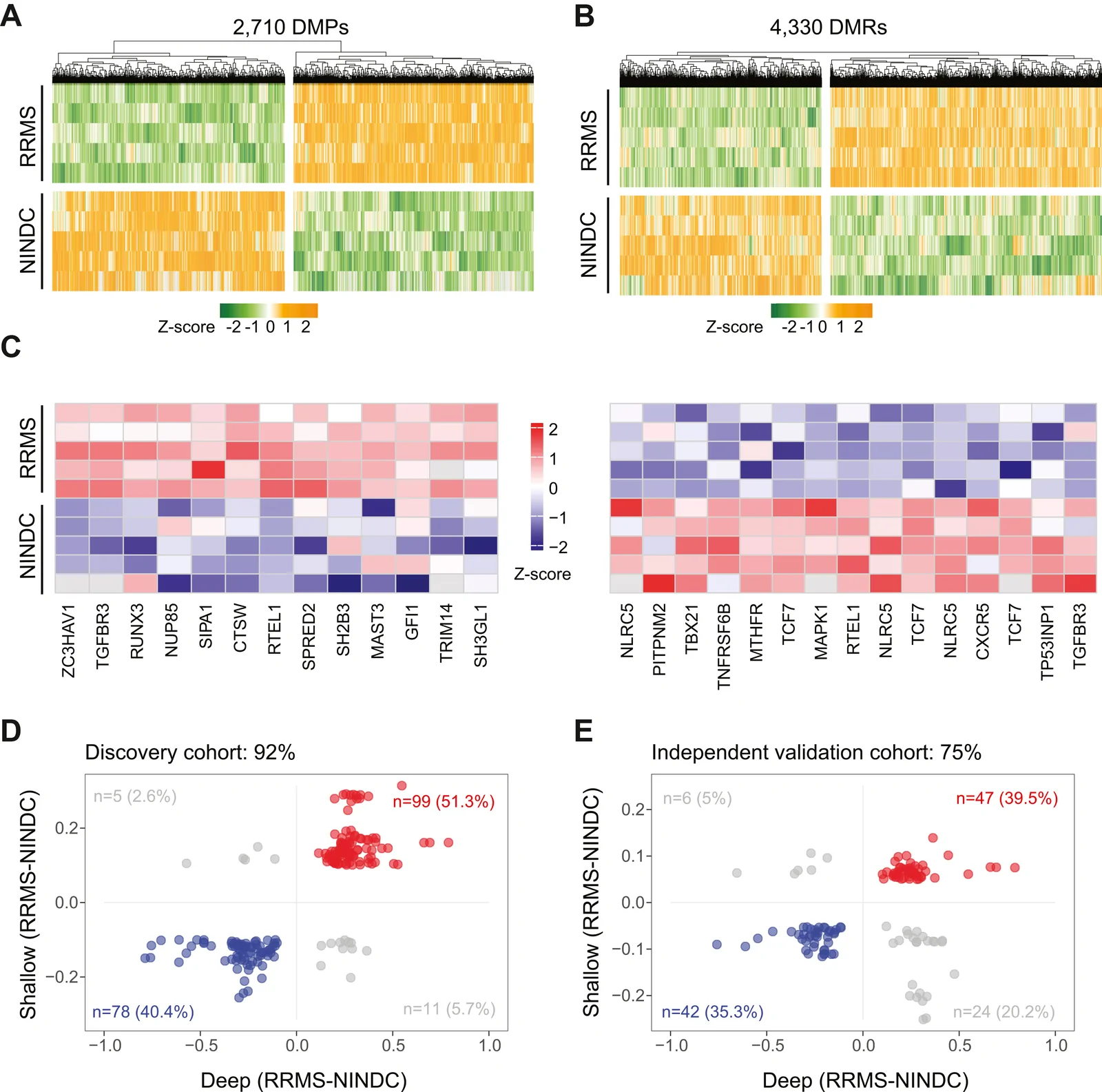
**Identification and replication of differential methylation in CSF cells from RRMS and NINDCs**. (**A-B)** Heatmap and hierarchical clustering of differentially methylated **(A)** positions (DMPs) and **(B)** regions (DMRs). The colour-key denotes Z-scored DNA methylation levels (green-to-orange). **(C)** Heatmaps of selected DMRs annotated to genes in RRMS and NINDC samples. Repeated gene names, where present, correspond to distinct DMRs annotated to the same gene and are displayed as separate rows. Colours indicate Z-scored DNA methylation levels, with red and blue representing higher and lower methylation, respectively. **(D**–**E)** Replication of DMPs in (D) the technical replication cohort, consisting of the same discovery cohort samples (n = 10), and (E) an independent validation cohort (n = 30), using shallow PBAT-seq. Colours indicate DMPs with the same direction of change in the discovery (deep PBAT-seq) and replication (shallow PBAT-seq) cohorts, with red illustrating increased and blue decreased DNA methylation levels in RRMS. Grey points represent DMPs changing in opposite directions. Number of DMPs and (percentages, %) are given in either category in their respective quadrant. DMP: differentially methylated position, DMR: differentially methylated region, RRMS: Relapsing–remitting multiple sclerosis, NINDC: non-inflammatory neurological disease controls.

To explore DNA methylation differences between RRMS and NINDC, we applied three complementary analytical methods, Limma, MethylKit, and RADmeth. Each method is built on distinct statistical principles and therefore identified a different number of differentially methylated positions (DMPs), the top significant CpGs were shared, i.e., top 1000 DMPs of each analysis were enriched in the other two methods (Supplementary Fig. S2A). In total, 2710 DMPs were detected with absolute methylation difference ≥10% and *P* < 0.001 by minimum two different ways of analysis (Supplementary Fig. S2B, Supplementary Table S4). The absolute methylation differences across these DMPs reached up to 89%, with a median of 21% (IQR: 15–29%). Heatmap and hierarchical clustering of DMPs clearly segregated RRMS from controls, revealing both hyper- and hypomethylated CpGs in RRMS (Fig. 2A).

To increase the robustness of detecting methylation changes with greater functional impact, we next identified differentially methylated regions (DMRs) by clustering CpG sites with a *P* < 0.01 and an absolute methylation difference ≥0.1 (see Methods and Supplementary Fig. S2B for details). In total, 4330 DMRs were identified (Fig. 2B, Supplementary Table S5), with the majority (74%) displaying hypermethylation in RRMS (Fig. 2B) and typically spanning < 1 kb in length (Supplementary Fig. S2C). The absolute methylation differences across these DMRs reached up to 75%, with a median of 17% (IQR: 15–20%). Methylation levels at the overlapping DMPs and DMRs were highly correlated (ρ > 0.9, Supplementary Fig. S2D and E).

To assess the relevance of identified changes to multiple sclerosis (MS), we overlapped DMPs and DMRs with reported MS risk genes.^5^ DMRs were significantly enriched within 2 kb of MS risk loci (permutation test, *P* = 0.033), with 70 of 560 candidate MS risk genes overlapping DMRs, among which 43 exhibited promoter methylation changes. Gene Ontology analysis of these 70 genes revealed enrichment for pathways related to cytokine responses, regulation of cell adhesion, and T helper (Th) cell differentiation (Supplementary Table S6). Further inspection indicated that many of these genes encode chemokines, interleukins, or their receptors, and approximately 30 of the 70 genes have established roles in T cell activation, regulation, and Th differentiation, including processes influencing the balance between Th17 and regulatory T cells (Treg) (Supplementary Table S6). After selecting genes with DMRs located in the promoter or flanking regions, we found genes associated with pro-inflammatory Th1/Th17 or activated T cell functions tended to be hypomethylated in MS, including T-box transcription factor 21 (*TBX21*) and C-X-C chemokine receptor type 5 (*CXCR5*), whereas genes linked to Treg or anti-inflammatory functions, such as interleukin-2 receptor subunit beta (*IL2RB*), were more frequently hypermethylated (Fig. 2C, Supplementary Table S6). When multiple DMRs mapped to the same gene, they were displayed separately rather than collapsed into a single gene-level value, as their methylation patterns may differ, and their functional relevance remains uncertain. In addition, one DMR (GRCh38: chr19:49,341,982–49,343,506) overlaps a polygenic risk score (PRS)–associated MS risk SNP (chr19:49,343,207) near CD37, a known negative regulator of T cell proliferation (Supplementary Fig. S3A).^64^

In the validation cohort, methylation profiles obtained at lower sequencing coverage were smoothed to reduce stochastic noise and improve methylation estimation accuracy^50^ (Supplementary Fig. S3B and C). The technical replicates validated 91.7% of testable DMPs, when applying a 10% methylation difference threshold (Fig. 2D) and 81.4% with a 5% cutoff (Supplementary Fig. S3D). Importantly, biological replication in an expanded independent cohort further confirmed consistent methylation differences for 74.8% of DMPs showing ≥5% methylation change (Fig. 2E, Supplementary Fig. S3A and B), a proportion significantly exceeding random expectation (Chi-square test, *P* = 6.9 × 10^−5^), supporting the robustness of the identified methylation signatures across cohorts.

In summary, integration of results from multiple statistical frameworks and validation in independent samples revealed widespread and reproducible genome-wide DNA methylation alterations in CSF cells from pwMS.

### DNA methylation changes highlight altered T cell adhesion and migration in pwMS

**Fig. 3:**
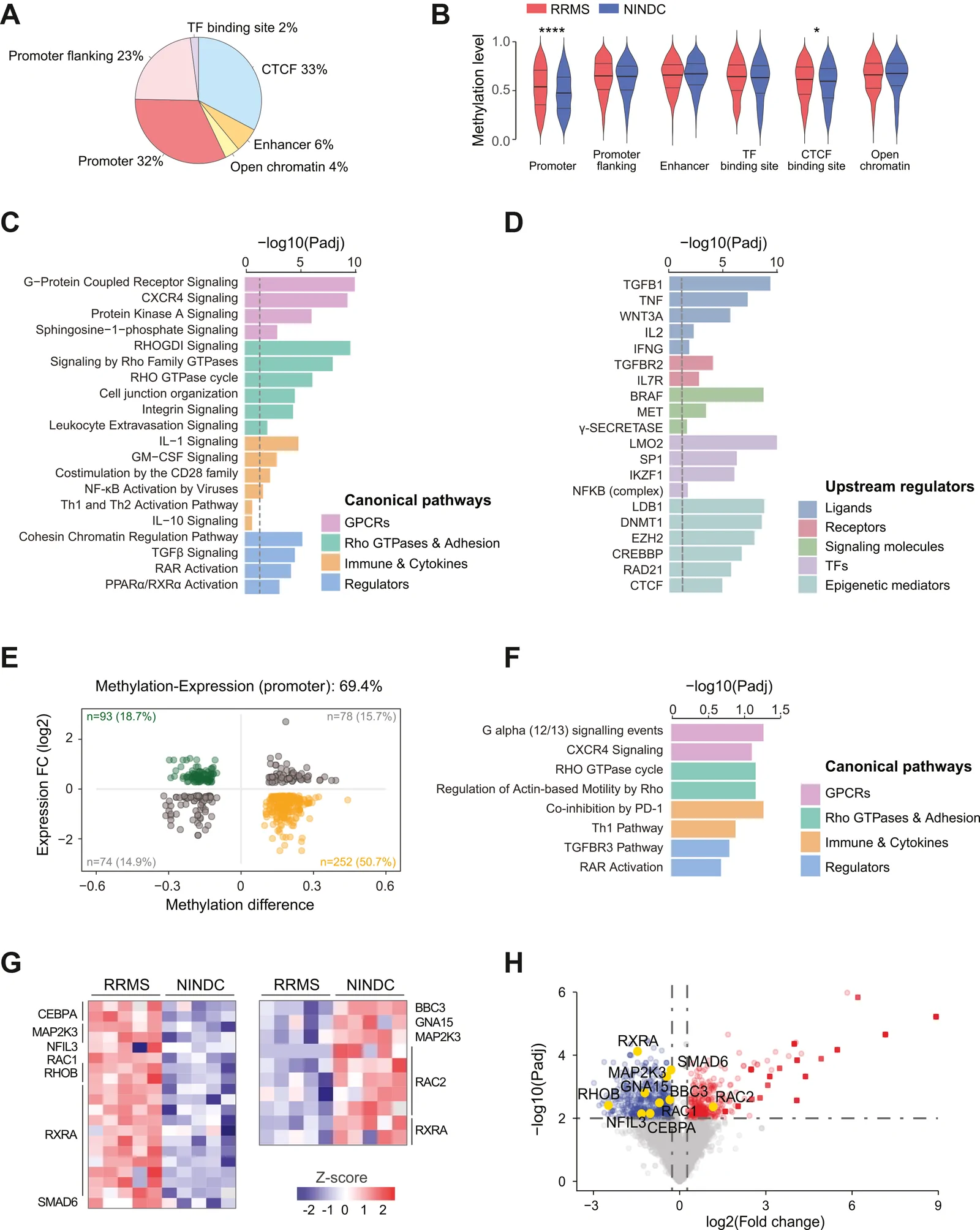
**Characteristics of differential methylation and downstream impact on RNA expression**. **(A)** Circle plot illustrating the percentage (%) of DMRs across genomic and regulatory features. **(B)** Distribution of DMR methylation levels across genomic and regulatory features in RRMS and NINDC samples. *P* values were calculated using two-sided Wilcoxon rank-sum tests and adjusted for multiple comparisons within each feature category using the Benjamini–Hochberg method. The central line indicates the median, and the upper and lower lines represent the 75 and 25^th^ percentiles, respectively. ∗: *P*adj < 0.05, ∗∗: *P*adj < 0.01, ∗∗∗∗: *P*adj < 0.0001. **(C**–**D)** Representative enriched pathways **(C)** and upstream regulatory factors **(D)** associated with differentially methylated genes. Bar length indicates enrichment significance (−log10 Padj) and colours represent different canonical pathways. GPCRs: G protein-coupled receptors. **(E)** Methylation differences of promoter-associated DMPs and DMRs (RRMS-NINDC) versus the log_2_ fold changes of corresponding Affymetrix gene expression levels. Genes are categorised according to concordant or inverse methylation–expression changes, in their respective quadrants with numbers (n) and percentages (%) indicated. Green dots indicate genes showing promoter hypomethylation accompanied by increased expression, whereas yellow dots represent genes with promoter hypermethylation and reduced expression. **(F)** Representative canonical pathways enriched among genes exhibiting inverse changes between promoter methylation and gene expression in MS samples. **(G)** Heatmaps of hypermethylated and hypomethylated DMP and DMR-associated genes involved in immune-related and regulatory pathways (from C) across RRMS and NINDC samples. **(H)** Volcano plot of log_2_ fold changes from Affymetrix data, with immune- and regulator-related genes highlighted and labelled. Red and blue dots indicate genes up- or downregulated expression in RRMS (|Foldchange| ≥ 1.2 and Padj <0.01). Red squares indicate immunoglobulin (IG) genes. DMR: differentially methylated region, DMP: differentially methylated position, RRMS: relapsing–remitting multiple sclerosis, NINDC: non-inflammatory neurological disease controls. Prom: promoter, Prom flk: promoter flanking region, Enh: enhancer, TFbs: transcription factor binding sites, CTCFbs: CTCF binding sites, Open: open chromatin. Dashed line indicates Padj = 0.05 significance threshold.

To further characterise MS-associated DMPs and DMRs, we analysed their distribution and methylation levels across CpG contexts and genomic features. Notably, MS samples exhibited increased methylation in CpG islands, CpG shores, open sea regions, CTCF binding sites, and promoter regions, suggesting a potential repressive effect on gene expression (Fig. 3A and B, Supplementary Fig. S4A–C). Notably, while most genomic features showed balanced distributions of hyper- and hypomethylated DMRs, promoter regions displayed a marked enrichment of hypermethylated DMRs in RRMS (Fig. 3B). We then used Ingenuity Pathway Analysis (IPA) to explore pathways of genes affected by methylation changes. The most significant pathways clustered into four categories: G protein-coupled receptors (GPCRs), Rho GTPases/adhesion, Immune/cytokines, and Regulators (Fig. 3C, Supplementary Tables S7 and S8). GPCRs represent a large family of cell surface receptors that transmit extracellular signals into the cell via activation of heterotrimeric G proteins, making them attractive targets for drug development.^65^ Interaction of C-X-C motif chemokine receptor type 4 (CXCR4), one of the GPCRs, with its ligand C-X-C motif chemokine ligand 12 (CXCL12) has been reported to guide chemotaxis of T cells and other immune cells, promoting their transmigration across the blood–brain barrier (BBB) and subsequent CNS infiltration in MS.^66^, ^67^, ^68^ In addition, the GPCR C–C motif chemokine receptor type 7 (*CCR7*) displayed promoter hypomethylation in pwMS (Supplementary Table S7). By primarily mediating immune cell responses from the C–C motif chemokine ligands CCL19 and CCL21, it directs naive and memory T-helper (Th) cells entry into lymph nodes for priming and has been implicated in shaping CNS-directed immune responses in MS.^69^ Consistently, CCR7 and its ligands are expressed both in peripheral lymphoid tissues and within the CNS in mice with experimental autoimmune encephalomyelitis (EAE).^70^ Pathways with ≥10 molecules and BH-adjusted *P* < 0.001 were considered in GeneSetCluster2,^54^ which identified two main clusters represented by cell adhesion and cell junction assembly (Supplementary Fig. S4D, Supplementary Table S8). Furthermore, predicted upstream regulators of differentially methylated genes included IL-2, TNF, IFNγ, and TGFB1/TGFBR2, among others (Fig. 3D, Supplementary Table S9).

To investigate the functional consequences of altered methylation in pwMS, we examined transcription using two datasets: an RNA-seq dataset generated from CSF cells^29^ and an independent Affymetrix microarray dataset.^28^^,^^63^ The RNA-seq dataset comprised 17 individuals (11 with RRMS and 6 NINDC). Of these, 5 individuals (4 RRMS and 1 NINDC) belonged to the discovery cohort, whereas the remaining 12 (7 RRMS and 5 NINDC) belonged to the independent replication cohort (Fig. 1A, Supplementary Table S3). Approximately 69.4% of DMPs and DMRs at promoter regions exhibited inverse methylation and expression changes (Supplementary Fig. S5A). More specifically, 47.8% of DMPs and 57.6% of DMRs exhibited hypermethylation and corresponding decrease in gene expression in pwMS (Supplementary Fig. S5A). We next analysed Affymetrix microarray data from CSF cells from an independent larger cohort of 26 RRMS and 18 NINDC,^28^^,^^63^ and could confirm consistent association between promoter hypermethylation and reduced gene expression in pwMS for nearly half of the altered genes (Supplementary Fig. S5A). The genes with inverse promoter methylation and expression changes were enriched in similar functional categories as the genes associated with DMPs and DMRs (Fig. 3E and F). Considering jointly RNA-seq and Affymetrix datasets, 164 genes displayed expression and methylation differences in CSF of pwMS (3.7% of 4461 genes with DMP or DMR) (Supplementary Fig. S5B). Most of these genes displayed hypermethylation and reduced expression. Notably, 12 were related to immune and regulatory pathways, 10 of which overlapped with DEGs identified by both RNA-seq and Affymetrix analyses (Fig. 3G and H and Supplementary Fig. S5C). For example, Retinoid X Receptor α (*RXRA*) gene contained multiple hypermethylated sites (Fig. 3G) and showed a pronounced reduction in expression in RRMS (Fig. 3H). In contrast, rac family small GTPase 2 *(RAC2)* was markedly upregulated in RRMS and exhibited multiple hypomethylated CpGs (Fig. 3G and H).

Collectively, these findings indicate that genes involved in T cell adhesion and migration exhibit coordinated changes in both DNA methylation and expression levels in pwMS.

### Differential MS methylation is enriched in active enhancers of memory T cells

**Fig. 4:**
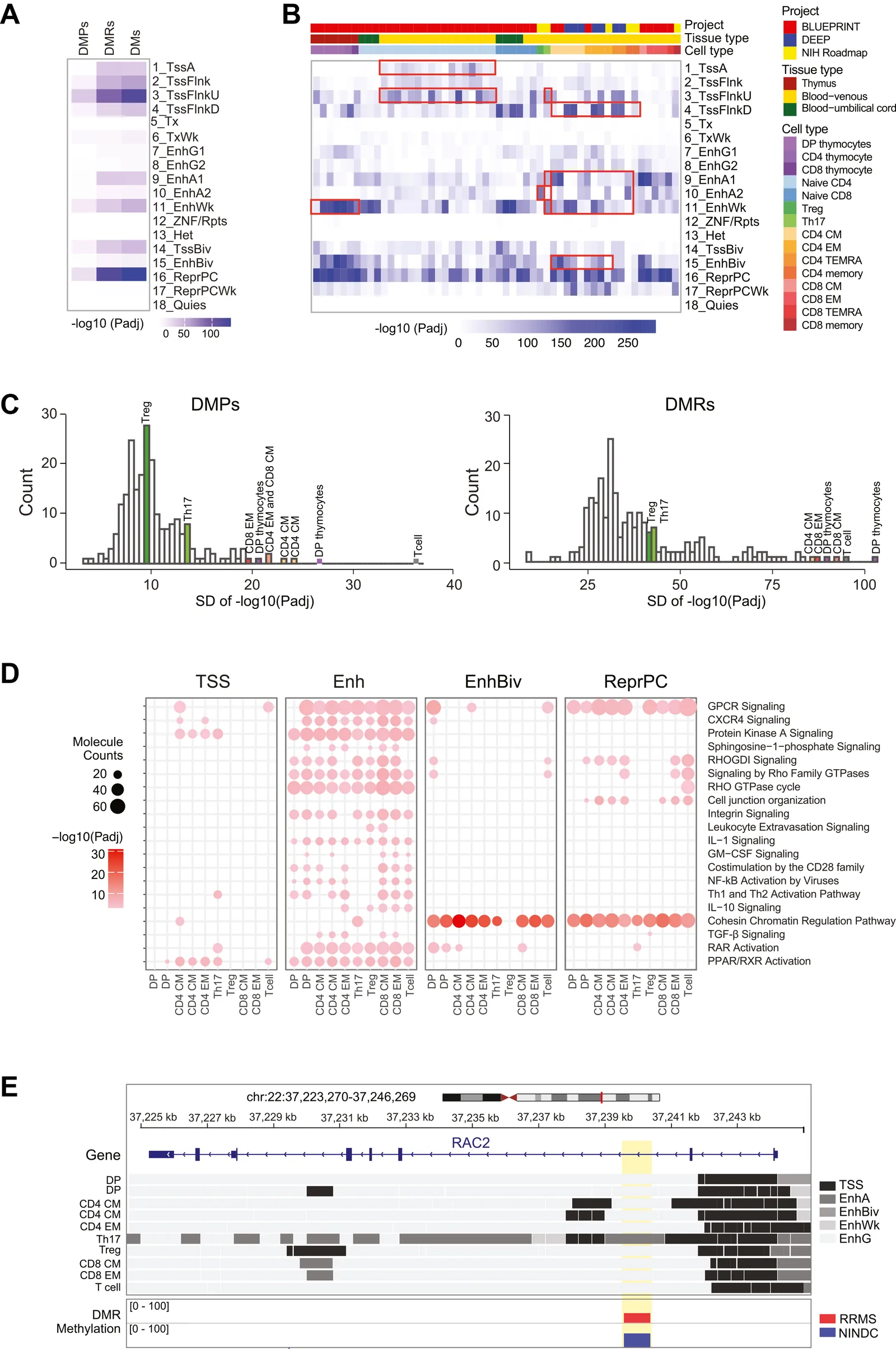
**Differential enrichment of DMPs and DMRs across T cell-derived chromatin states**. **(A**–**B)** Heatmaps showing enrichment of **(A)** DMPs and DMRs across summary T cell chromatin states and **(B)** DMRs across selected, representative T cell subtype samples. The colour intensity (white-to-blue) represents enrichment significance, −log10(Padj). Top annotation bars indicate project, tissue type, and cell type. Red boxes highlight regions with enrichment differences of interest. **(C)** Distribution of the standard deviation (SD) of enrichment −log10(Padj) values across all T cell-related chromatin states overlapping with DMPs and DMRs, indicating variability in feature enrichment across T cell subsets. Coloured bars and labels denote T cell subtypes with high SD and of particular interest. **(D)** Representative IPA pathway enrichment across selected T cell subsets within four combined chromatin states: transcription start site (TSS), enhancer (Enh), bivalent enhancer (EnhBiv), and Polycomb-repressed regions (ReprPC). Dot size indicates molecule counts and colour intensity (white-to-red) reflects enrichment significance, −log10(Padj). **(E)** A representative genomic view of the *RAC2* locus showing localisation of DMPs and DMRs (highlighted in yellow) relative to chromatin states across summary and selected T cell subsets. DMP: differentially methylated position, DMR: differentially methylated region, DM: DMPs plus DMRs, 1_TssA: Active transcription start site, 2_TssFlnk: Flanking transcription start site, 3_TssFlnkU: Upstream flanking transcription start site, 4_TssFlnkD: Downstream flanking transcription start site, 5_Tx: Strong transcription, 6_TxWk: Weak transcription, 7_EnhG1: Genic enhancer 1, 8_EnhG2: Genic enhancer 2, 9_EnhA1: Active enhancer 1, 10_EnhA2: Active enhancer 2, 11_EnhWk: Weak enhancer, 12_ZNF/Rpts: ZNF genes and repeats, 13_Het: Heterochromatin, 14_TssBiv: Bivalent/poised transcription start site, 15_EnhBiv: Bivalent enhancer, 16_ReprPC: Polycomb-repressed region, 17_ReprPCWk: Weak Polycomb repression, 18_Quies: Quiescent/low signal region, DP thymocytes: Double-positive thymocytes, CD4 thymocyte: CD4^+^ thymocytes, CD8 thymocyte: CD8^+^ thymocytes, Naive CD4: Naive CD4^+^ T cells, Naive CD8: Naive CD8^+^ T cells, Treg: Regulatory T cells, Th17: T helper 17 cells, CD4CM: CD4^+^ central memory T cells, CD4EM: CD4^+^ effector memory T cells, CD4TEMRA: CD4^+^ terminally differentiated effector memory T cells re-expressing CD45RA, CD4 memory: CD4^+^ memory T cells, CD8CM: CD8^+^ central memory T cells, CD8EM: CD8^+^ effector memory T cells, CD8TEM: CD8^+^ terminally differentiated effector memory T cells re-expressing CD45RA, CD8 memory: CD8^+^ memory T cells.

To further characterise MS-associated methylation changes, we investigated potential cell type-specific regulation by leveraging cell type-specific chromatin states derived from IHEC (preprint, not peer-reviewed).^37^ Enrichment analysis across a 100-state universal chromatin model^71^ showed strongest enrichment in the bivalent promoter state 2 (BivProm2), as well as significant enrichment of DMPs and DMRs in EnhA8 and EnhA9, enhancer states typically associated with blood immune cells (Supplementary Fig. S6A). Given that CSF cells predominantly consist of T cells, we next examined an 18-state model, which was generated by IHEC using 168 samples comprising diverse T cell subsets. We found the highest enrichment in TSS, particularly upstream flanking (TSSFlnkU), regions repressed by Polycomb (ReprPC) and both active and weak enhancers (EnhA1 and EnhWk) in T cells (Fig. 4A). We further conducted enrichment analysis in 229 individual T cell samples from IHEC, demonstrating that while a significant enrichment of MS-associated DMRs was observed in ReprPC across most T cell subsets, more distinct cell type-specific patterns emerged in specific T cell subsets (Fig. 4B, Supplementary Fig. S6B and C). Specifically, in naive CD4^+^ T cells, MS-associated DMRs were predominantly enriched in active TSS (TSSA and TSSFlnkU), but in memory CD4^+^ T cell subsets they were mainly enriched in active and weak enhancers (EnhA1, EnhA2 and EnhWk) and downstream flanking TSS (TSSFlnkD) (Fig. 4B). Additionally, enrichment in bivalent enhancers (EnhBiv) was the most pronounced in effector and central memory CD4^+^ T cells. In Tregs, the most significant enrichment was in EnhA2, while in Th17 cells, EnhA1, EnhWk and TSSFlnkU were enriched in MS-associated DMRs. Highly significant enrichment was found in weak enhancers (EnhWk) of immature T cells from thymus and umbilical cord (Fig. 4B).

Based on the distribution of SD values calculated from −log10(Padj) enrichment values (Fig. 4C), the most heterogeneous enrichment patterns were observed in bulk T cells, double-positive T cells, central and effector memory CD4^+^ and CD8^+^ T cells. To explore potential functional impact of differential methylation in these subsets, including Treg and Th17 (Fig. 4C, Supplementary Fig. S6D), we next conducted IPA analysis on the differentially methylated genes mapping to specific chromatin states in a given T cell subset. We found that genes in proximity to enhancer-located MS differentially methylated sites were significantly enriched in pathways such as GPCR signalling, Rho GTPases cycle and RAR activation across most T cell subtypes (Fig. 4D), suggesting their expression modulation via enhancer methylation in pwMS. A subset of pathways was more specifically associated with promoter (TSS) regions especially in memory CD4^+^ T cells, including GPCR Signalling, PKA Signalling and PPAR/RXR activation (Fig. 4D). Interestingly, the Cohesin Chromatin regulation pathway demonstrated particularly strong enrichment in EnhBiv and ReprPC regions across all subsets, and active enhancers in Th17 specifically (Fig. 4D). While some genes were shared across multiple samples and states, most were uniquely associated with specific cell types and/or chromatin states (Supplementary Fig. S7). This is exemplified by the regulatory context of *RAC2*, where DMPs and DMRs reside in genic enhancer (EnhG) regions in bulk T cells and most other cell types, but transition to active enhancer states in Th17 cells, highlighting differentiation state-specific epigenetic regulation (Fig. 4E).

Our integrative analysis of MS-associated differential methylation with T cell subset-specific chromatin states suggests a predominant role of enhancer, and to a much lower degree promoter, methylation in regulating T cell activation and migration in pwMS.

### Protocadherin genes are epigenetically repressed in pwMS and implicated in immune regulation

**Fig. 5:**
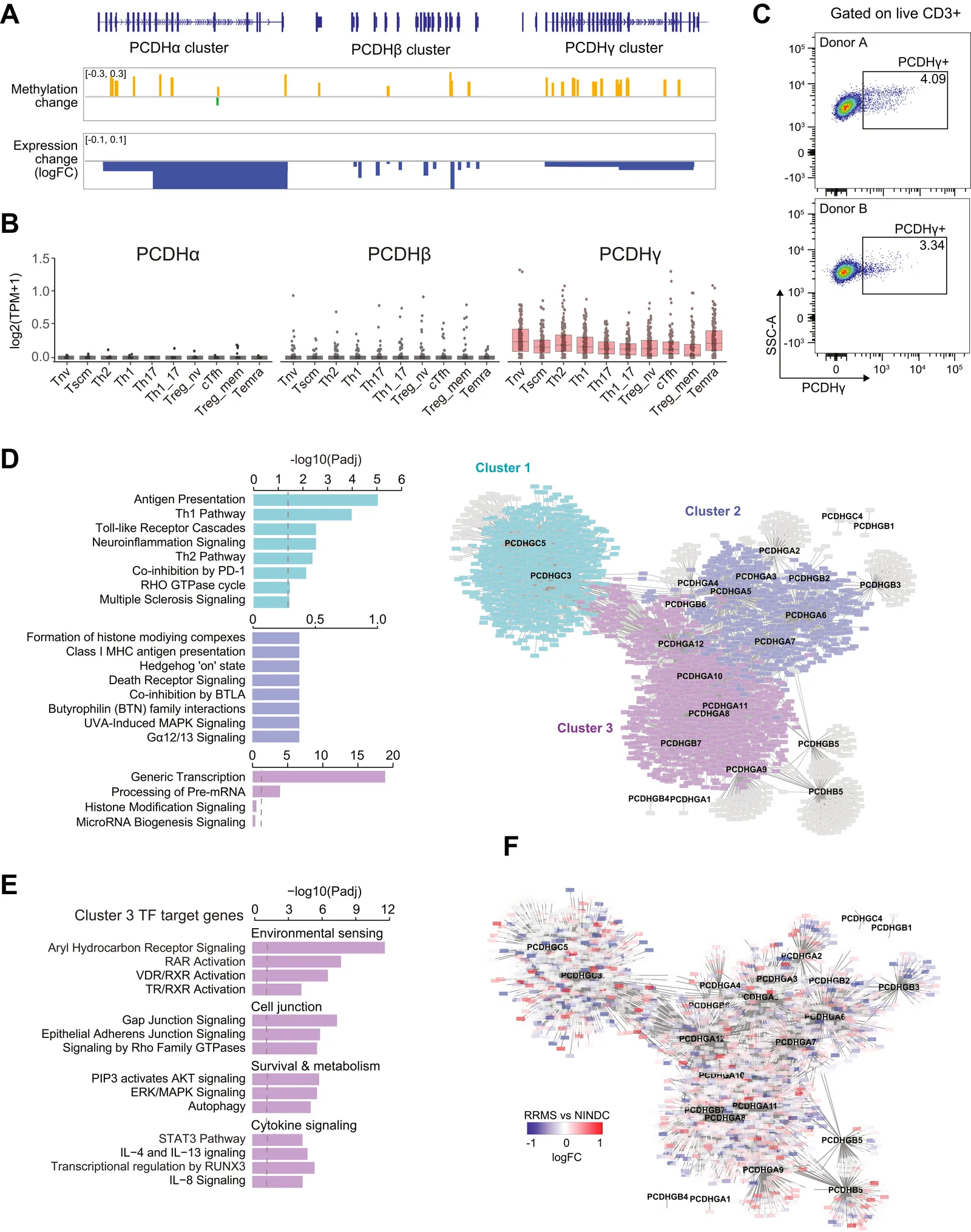
**MS-associated alterations of PCDH loci and PCDH expression in human T cells**. **(A)** DNA methylation differences (RRMS-NINDC) and gene expression changes (log_2_ fold change) across protocadherin (PCDH) gene functional modules. Orange and green bars represent increased and decreased DNA methylation, respectively, and blue bars indicate gene downregulation (logFC). **(B)** Expression patterns of *PCDHα*, *PCDHβ*, and *PCDHγ* clusters across ten T cell subsets. Expression values are shown as log_2_ (TPM+1). Boxplots show median and interquartile range, points indicate individual samples. **(C)** Representative flow cytometry plots showing PCDHγ expression in gated live CD3^+^ T cells from two healthy donors. Cells were analysed by PCDHγ versus side scatter (SSC-A), with PCDHγ^+^ cells indicated. Percentages represent the proportion of PCDHγ^+^ cells among CD3^+^ T cells. **(D)** Co-expression network of PCDH-associated genes constructed using Spearman correlation. Functional enrichment of major gene clusters is shown on the left. Edges represent significant correlations (|r| ≥ 0.5 and Padj <0.01). Nodes and pathways are coloured according to cluster membership. **(E)** Representative functional categories enriched among transcriptional targets associated with Cluster 3 genes. **(F)** PCDH co-expression network with nodes coloured according to pseudobulk RRMS versus NINDC log_2_ fold changes in CSF CD4^+^ T cells from single-cell RNA-seq data. PCDH: protocadherin. FSC-A: forward scatter area, SSC-A: side scatter area. RRMS: relapsing remitting multiple sclerosis, NINDC: non-inflammatory neurological disease controls. Dashed line indicates Padj = 0.05 significance threshold.

While methylation changes in pwMS were distributed genome-wide, a considerable aggregation of changes was observed in Protocadherin (*PCDH*) genes and clusters, with 6 DMPs and 27 DMRs affecting 43 distinct *PCDH* genes (Fig. 5A, Supplementary Tables S4 and S5). Protocadherin genes constitute a large family of cell-adhesion molecules, categorised into clustered (α, β, γ) and non-clustered genes. They are conventionally expressed in the nervous system to regulate neuronal adhesion, connectivity, and circuit formation,^72^ while their role in immune cells remains largely unexplored. We observed extensive methylation alterations across multiple *PCDH* genes, with all DMPs and DMRs except one showing hypermethylation in RRMS compared to controls (Fig. 5A). Consistently, transcriptomic data revealed modest but widespread reduction of the expression of multiple *PCDH* genes in RRMS (Fig. 5A). Moreover, *PCDH* genes were the main component of the *Cohesin chromatin-regulation pathway* that demonstrated striking enrichment of MS-associated methylation changes across T cell subsets and Th17 cells in particular (Fig. 4D).

Because PCDH genes are conventionally associated with neuronal expression and function, we next asked whether these genes are expressed in human T cells. For this analysis, we used RNA-seq data from sorted T cell subsets from healthy individuals; this analysis was not designed as an MS-control comparison. Across these subsets, expression of the *PCDH*α and *PCDH*β cluster genes, as well as non-clustered *PCDHs*, was generally low, whereas the *PCDH*γ cluster genes showed appreciable expression (Fig. 5B). Notably, *PCDH*γ genes were most expressed in naive T cells (Tnv) and terminally differentiated effector memory T cells re-expressing CD45RA (TEMRA) (Fig. 5B). To further validate *PCDH*γ expression at the protein level, we performed intracellular flow cytometry on peripheral blood T cells using an anti-PCDHγ (pan) C-terminal antibody that cross-reacts with *PCDH*γ-A, -B, and –C isoforms. We detected intracellular PCDHγ protein in 3–4% on average of live T cells (Fig. 5C, Supplementary Fig. S8A and B), supporting a potential role for *PCDH*γ genes in T cell biology. This flow cytometry panel was used to demonstrate detectability of PCDHγ protein in human T cells and does not represent an MS-control comparison.

To investigate potential functional roles, we performed co-expression analysis between *PCDH* genes and other genes in the T cell subsets. For descriptive interpretation, network genes were organised into three major functional modules based on their connectivity to distinct PCDH genes, T-cell-subset expression patterns, and IPA functional enrichment results. Cluster 1 predominantly comprised first neighbours of *PCDHGC3* and *PCDHGC5*, which showed the highest expression in terminally differentiated effector memory T cells (TEMRA) (Fig. 5D, Supplementary Fig. S9). Genes within this cluster were enriched in pathways related to T cell activation and differentiation (Fig. 5D, Supplementary Table S10). Cluster 2 mainly consisted of first neighbours of *PCDHGA5-7* whose function is enriched in activation, chromatin and cell cycle related pathways (Fig. 5D, Supplementary Fig. S9, Supplementary Table S10). Cluster 3 was primarily driven by first neighbours of *PCDHGA8,10–11* and *PCDHGB7*, which exhibited relatively lower abundance in Th1/Th17 cells (Fig. 5D, Supplementary Fig. S9). This cluster contained a substantial number of TFs, resulting in the strong enrichment of the transcription and mRNA processing pathways (Supplementary Table S10). Examination of TF target genes expressed in T cells further revealed pathways such as *Aryl Hydrocarbon Receptor Signalling*, *RAR Activation*, *VDR*/*RXR Activation*, *mTOR Signalling*, among others (Fig. 5E, Supplementary Table S10). Genes co-expressed with *PCDH* genes in the Affymetrix dataset were enriched for *cAMP-mediated signalling* and *G protein–coupled receptor signalling pathways* (Supplementary Fig. S8C).

Finally, to relate the PCDH co-expression network back to disease-associated transcriptional changes, we projected RRMS versus control gene expression log2 fold changes from CSF CD4^+^ T cells onto the co-expression network. Genes co-expressed with PCDHs showed an overall tendency towards reduced expression in RRMS, although this pattern was not uniform across all network components (Fig. 5F). Together, these analyses indicate that *PCDH* loci show MS-associated methylation and expression changes in CSF cells, while *PCDHγ* transcripts and protein are detectable in human T cells and are linked to transcriptional programmes relevant to T cell activation and immune regulation.

### PCDHγ expression in CD4^+^ T cells is altered in MS

**Fig. 6:**
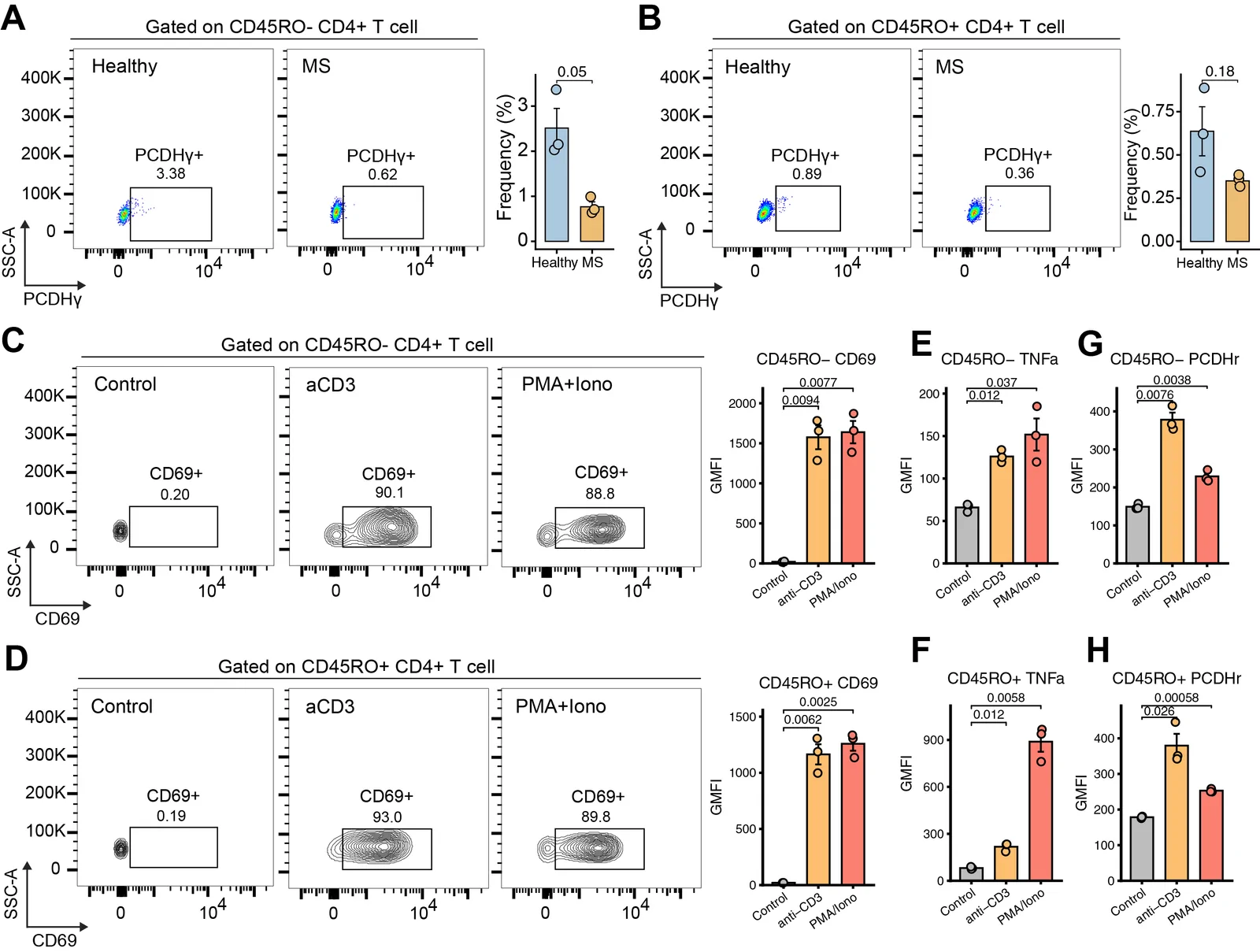
**Flow cytometric validation of PCDHγ protein expression and stimulation-induced activation in CD4^+^ T-cell subsets**. **(A–B)** Representative flow cytometry plots and quantification of PCDHγ-positive cells in CD45RO^−^ CD4^+^ T cells (A) and CD45RO^+^ CD4^+^ T cells (B) from healthy controls and pwMS. **(C–D)** Representative flow cytometry plots showing CD69 induction in CD45RO^−^ CD4^+^ T cells (C) and CD45RO^+^ CD4^+^ T cells (D) from MS samples under unstimulated control, PMA/ionomycin, and anti-CD3 stimulation conditions. (E–H) Quantification of geometric mean fluorescence intensity (GMFI) for CD69, TNFα, and PCDHγ in CD45RO^−^ CD4^+^ T cells **(E, G)** and CD45RO^+^ CD4^+^ T cells **(F, H)** from MS samples after stimulation. Bars show mean ± SEM; each dot represents an individual donor after averaging technical replicates. *P* values in A-B were calculated using unpaired t-tests comparing healthy controls and pwMS. *P* values in E-H were calculated using paired t-tests comparing stimulated conditions with unstimulated controls.

We next extended the flow cytometry analysis to PBMCs from healthy donors and pwMS and examined pan-PCDHγ expression in CD45RO− and CD45RO^+^ CD4^+^ T cell subsets. The gating strategy used to identify PCDHγ^+^ cells within CD45RO− and CD45RO^+^ CD4^+^ T cell subsets is shown in Supplementary Fig. S10A. The frequency of pan-PCDHγ^+^ cells was lower in pwMS compared with healthy donors in CD45RO^−^ CD4^+^ T cells (*P* = 0.05), with a similar but non-significant trend observed in CD45RO^+^ CD4^+^ T cells (*P* = 0.18) (Fig. 6A and B). These data suggest reduced PCDHγ protein expression in circulating CD4+ T cells from pwMS, most prominently in the CD45RO− subset, consistent with the direction of the methylation and RNA-seq findings.

To determine whether PCDHγ expression is regulated by T cell activation, we stimulated PBMCs from pwMS donors with anti-CD3 for 24 h and with PMA/ionomycin during the final 6 h of a 24 h culture. Both stimulation conditions induced strong upregulation of the activation marker CD69 and TNFα in CD45RO^−^ and CD45RO^+^ CD4^+^ T cells, confirming efficient T cell activation (Fig. 6C–F, Supplementary Fig. S10B and C). In contrast, pan-PCDHγ expression showed a distinct pattern: anti-CD3 stimulation increased PCDHγ GMFI in both CD45RO− and CD45RO^+^ CD4^+^ T cells, whereas PMA/ionomycin resulted in lower PCDHγ expression compared with anti-CD3 stimulation (Fig. 6G and H). Together, these findings indicate that PCDHγ expression in CD4^+^ T cells is activation-responsive but depends on the mode of stimulation, supporting a potential role for PCDHγ in T cell activation states relevant to MS.

## Discussion

In this study, we present a comprehensive DNA methylation analysis of CSF cells. Our findings reveal distinct CSF methylation patterns in pwMS with numerous reproducible differentially methylated regions compared to other neurological disease controls. These alterations were predominantly enriched in pathways related to cytokine signalling, immune activation, and cell adhesion and migration, and were accompanied by corresponding changes in RNA expression. Interestingly, abundant methylation changes affected *PCDH* genes and clusters, highlighting a previously unrecognised potential role of protocadherins in pwMS, and leading to plausible mechanistic connections that may link environmental cues to immune regulation.

Consistent with the preserved global DNA methylation architecture observed in CSF cells, we did not detect significant alterations in the expression of members of the DNMT and TET family, *UHRF1* and *UHRF2*, that would indicate broad transcriptional dysregulation of the methylation machinery. Instead, the MS-associated methylation changes likely arise from locus-specific regulatory remodelling shaped by immune-cell activation state, chromatin context, transcription factor recruitment, which could in part be driven by the genetic predisposition and/or environmental exposures.

We identified numerous methylation changes in regulatory regions of genes strongly enriched in GPCR signalling, Rho GTPase pathways, cytokine signalling, and regulators of immune activation, suggesting enhanced migratory potential and altered communication of CSF immune cells in pwMS. Since we focused on methylation differences larger than 10%, it is unlikely that our findings are driven by bias in cell type composition, as our deconvolution results (Fig. 1E), together with single-cell and flow cytometry analyses, indicate that differences in immune cell proportions between MS and controls are substantially smaller than this threshold.^32^^,^^33^ Instead, the observed methylation changes likely reflect intrinsic epigenetic alterations within T cells that constitute on average 90% of CSF cells. Our findings, at the epigenetic level of regulation, reinforced the support for enhanced migratory and adhesive properties of immune CSF cells, which may facilitate their ability to cross the BBB and are consistent with the established roles of GPCRs in promoting T cell chemotaxis, BBB transmigration, and CNS infiltration in MS.^67^^,^^73^ Importantly, by mapping these alterations across regulatory features, we show that they are not randomly distributed but are preferentially localised to enhancers of genes enriched in these pathways. This enrichment was particularly pronounced in memory CD4^+^ T cells, indicating that cells with a high migratory/inflammatory potential are epigenetically primed via specific regulations that are likely causal to corresponding expression changes instead of being a consequence of gene usage. These data uncover a cell type-specific and functionally oriented epigenetic architecture in which DNA methylation at regulatory regions contributes to cytokine signalling, GPCR–Rho GTPase pathways, adhesion, and migration programmes in CSF T cells of pwMS. As an example, we identified coordinated DNA methylation and transcriptional alterations in key regulators including *RAC2*, which showed promoter-associated methylation changes accompanied by increased expression in RRMS. *RAC2* encodes a small GTPase with an established role in cytoskeletal remodelling, T cell motility, and autoimmune inflammation.^74^^,^^75^ Importantly, a large fraction of genes showed concordant methylation and expression changes, with promoter hypermethylation generally associated with reduced gene expression in pwMS (Fig. 3E). Together, these results suggest that CSF pathogenic immune cells in MS are epigenetically programmed towards enhanced migration and adhesion, potentially supporting their accumulation within the CNS inflammatory niche.

In addition to alterations affecting genes and pathways involved in cell trafficking, DNA methylation changes in CSF immune cells converged on genes and pathways regulating the balance between pathogenic Th1/Th17 responses and regulatory T cell (Treg) programmes. Several genes implicated in pro-inflammatory T cell states displayed promoter hypomethylation in pwMS. Those include for example T-box transcription factor 21 *(TBX21)* gene,^76^ adhesion G protein-coupled receptor E5 *(ADGRE5)*,^77^ and interleukin-32 *(IL32)*^78^ (Supplementary Tables S4 and S5). *TBX21* encodes T-bet, a central regulator of Th1 differentiation and IFN-γ-associated effector responses in T cells. However, *TBX21* also has important functions in B cells. B-cell receptor, Toll-like receptor, and IFN-γ signalling can induce T-bet and promote atypical or age-associated B-cell phenotypes associated with chronic immune activation and autoimmunity.^79^, ^80^, ^81^ Consistent with these observations, our recent collaborative study demonstrated that T-bet^+^CXCR3^+^ B cells amplify and sustain Th1 responses through IFN-γ-dependent B-T-cell interactions in MS. These cells exhibited epigenetic and transcriptional activation, clonal evolution from memory B cells towards somatically hypermutated plasma blasts and were detected in highly inflamed MS meningeal tissue.^82^

Nevertheless, the *TBX21* methylation alteration detected in our bulk CSF-cell dataset is likely driven predominantly by T cells. T cells represented approximately 90% of CSF immune cells in our dataset, whereas the estimated difference in total B-cell abundance between the RRMS and control groups was less than 10% points. As DMP identification required an absolute methylation difference of at least 10%, the *TBX21* alteration is unlikely to be explained solely by this modest difference in B-cell composition. However, methylation changes occurring within T-bet^+^ B cells cannot be excluded, and cell-type-specific methylome or single-cell multi-omics profiling will be required to distinguish their contribution from T-cell-derived alterations.

Among the other hypomethylated genes, ADGRE5 encodes CD97, which is commonly expressed in activated and inflammatory T cells in MS,^77^ whereas IL32 encodes a pro-inflammatory cytokine reported to interact with IL-17 and characterise Th17 cells.^78^ Consistently, interleukin-1 receptor accessory protein (IL1RAP) was also hypomethylated in pwMS, along with significant enrichment of DMPs and DMRs across genes of *IL-1 programmes pathway* (Supplementary Tables S8 and S9). IL-1 signalling is a central driver of pathogenic T cell differentiation and CNS trafficking in MS, sustaining inflammatory activation and effector T cell responses.^83^ In contrast, several MS candidate genes with established immunoregulatory functions exhibited promoter hypermethylation in CSF cells of pwMS. These included transforming growth factor beta receptor 3 *(TGFBR3)*, which modulates TGF-β signalling and has been implicated in limiting Th17-type inflammatory responses in EAE,^84^ and interleukin-10 receptor subunit alpha *(IL1*0RA), which mediates IL-10 signalling, a central pathway in Treg-mediated immunoregulation (Supplementary Tables S7 and S8).^85^ This inflammatory bias may be partially counteracted by alterations in retinoic acid receptor–retinoid X receptor (RAR–RXR) signalling, with retinoic acid receptor alpha *(RARA)* displaying promoter hypermethylation in pwMS (Supplementary Tables S7 and S8), which has been reported to promote regulatory T cell differentiation and inhibit Th17 cell development.^86^

An intriguing finding was the widespread hypermethylation across multiple *PCDH* genes in pwMS, accompanied by coordinated reduction in gene expression in CSF immune cells, as protocadherins are primarily known for their roles in neuronal cell adhesion and their function in immune cells remains poorly understood. Consistent with our observations, independent studies of MS-discordant monozygotic twins have reported altered methylation across *PCDH* loci, including hypermethylation of the *PCDHγ* cluster and *PCDH10* in PBMCs of affected twins.^87^ The *PCDH* gene family of adhesion molecules is critical for neuronal connections and wiring.^88^ Various extracellular signals can converge on the same intracellular signalling pathway through the common CD domain shared by members of PCDHα and PCDHγ.^89^, ^90^, ^91^, ^92^ Mechanistically, isoforms of PCDHα and PCDHγ can be cleaved by γ-secretase and A disintegrin and metalloprotease (ADAM10), subsequently releasing the intracellular domain, which translocate into the nucleus and regulates gene expression.^93^, ^94^, ^95^, ^96^ In addition, the intracellular domains of PCDHα and PCDHγ can bind to several kinases involved in cytoskeleton dynamics and cell adhesion, including focal adhesion kinase (FAK), proline-rich tyrosine kinase 2 (Pyk2), and receptor tyrosine kinase rearranged during transformation (Ret).^91^^,^^97^, ^98^, ^99^, ^100^ Together, these findings suggest that protocadherin dysregulation may extend beyond neuronal contexts and contribute to immune-associated regulatory processes. Accordingly, non-clustered PCDHs have been reported to regulate T cell infiltration in cancers^101^^,^^102^ and display altered expression following EBV infection in B cells.^103^ Moreover, PCDHGA10 is among the targets of primed DNA loops that facilitate rapid gene activation in memory B cells (preprint, not peer-reviewed),^104^ while PCDHGA1 has been reported as a driver for cell ageing.^105^ Additional evidence linking protocadherin loci to immune-related processes comes from the identification of methylation changes in *PCDHB14* in relation to other inflammatory conditions.^106^ However, the expression and functional roles of protocadherins, particularly clustered PCDHs, in immune cells remain largely unexplored warranting future studies.

Extending these observations, our flow cytometry analysis showed that the frequency of pan-PCDHγ^+^ cells was reduced in CD45RO^−^ CD4^+^ T cells from pwMS, with a similar trend in CD45RO^+^ CD4^+^ T cells. This protein-level reduction is consistent with the coordinated *PCDH* hypermethylation and lower transcript levels observed in CSF immune cells, suggesting that PCDHγ dysregulation is not restricted to the CSF compartment but may also be detectable in circulating CD4^+^ T cells. Although the sample size was limited, the stronger difference in CD45RO^−^ CD4^+^ T cells may indicate that altered PCDHγ expression emerges already in less differentiated CD4^+^ T cells or reflects changes in their activation potential. In vitro stimulation further indicated that PCDHγ expression is dynamically regulated by T cell activation. Anti-CD3 stimulation increased PCDHγ protein levels in both CD45RO^−^ and CD45RO^+^ CD4^+^ T cells, whereas PMA/ionomycin, despite inducing robust CD69 and TNFα responses, did not produce the same increase. This suggests that PCDHγ expression is not simply a generic consequence of strong T cell activation but may be more closely linked to TCR-proximal signalling or specific activation states. These findings support the idea that protocadherins may participate in immune-cell regulatory programmes, potentially related to T cell activation, adhesion, or signalling, rather than being passive markers of neuronal lineage-associated gene expression.

Supporting a functional role in immune cells, we observed close co-expression between PCDH genes and transcriptional regulators, and flow cytometry confirmed intracellular PCDHγ expression in T cells. Furthermore, γ-secretase emerged as a predicted upstream regulator of differentially methylated genes in our analysis (Fig. 3D). Distinct expression patterns of γ-secretase subunits were observed across T cell subsets (Supplementary Fig. S8D). In addition, we observed downregulation of one γ-secretase subunit, presenilin 1 (PSEN1, Padj <0.05, FC ≤ −1.2), in pwMS. These observations collectively suggest altered regulation of γ-secretase-mediated signalling and potential downstream transcriptional effects linked to protocadherins. Interestingly, MS-associated methylation changes were enriched in the *Cohesin Chromatin regulation pathway*, in which *PCDH* genes constituted a substantial component. Given that cohesin is a key mediator of chromatin looping and its anchoring is regulated by CTCF, which functions as a boundary element constraining enhancer–promoter interactions, altered methylation at CTCF motifs may disrupt regulatory domain organisation. Interestingly, the enrichment of the cohesin pathway was most pronounced with differentially methylated positions residing in enhancers of Th17 cells, contrasting with preferential localisation at bivalent enhancers in all T cell subsets except Tregs. Together, this suggests that dysregulation of *PCDH* may be linked to epigenetic perturbation of the cohesin-CTCF axis and altered 3D regulatory networks in pro-inflammatory T cells, particularly in Th17 encephalitogenic cells.

These observations jointly support a model in which epigenetic dysregulation of protocadherin loci, altered γ-secretase signalling, and perturbation of higher-order chromatin organisation converge to reshape transcriptional programmes in T cells in multiple sclerosis. Interestingly, the pathways targeted by those transcriptional regulators were enriched for *Aryl hydrocarbon receptor (AHR) signalling pathway*, with *AHR* displaying hypermethylation and AHR repressor (*AHRR*) exhibiting promoter hypomethylation in pwMS. AHR is a ligand-activated transcription factor residing in the cytoplasm under resting conditions that translocate to the nucleus upon activation and regulate gene expression. AHR ligands are diverse and include xenobiotics present in environmental pollutants, endogenous metabolic products such as tryptophan derivatives, and microbiota- or diet-derived metabolites, making AHR a key player in the integration of environmental and metabolic signals into immune regulation.^107^ AHR can direct T cell differentiation towards either Treg or Th17 lineages in response to distinct ligands which can consequently ameliorate or exacerbate MS-like disease in mice.^108^^,^^109^ Importantly, consistent with *AHRR* promoter hypomethylation observed in CSF cells of pwMS, smoking, known to predispose both to MS development and to more severe disease, induces pronounced hypomethylation and transcriptional upregulation of the *AHRR* gene in blood immune cells of pwMS.^110^ Together, these observations suggest that PCDH-associated transcriptional programmes converge on AHR signalling that intersects environmental, metabolic, and viral cues (including EBV activity) and influences immune regulation and potentially contributes to MS pathogenesis.

More broadly, our findings are consistent with previous peripheral immune-cell methylation studies in MS, which have repeatedly implicated immune regulatory loci such as the HLA region, particularly *HLA-DRB1*, as well as methylation differences across CD4^+^ T cells, CD8^+^ T cells, B cells, and monocytes.^14^^,^^15^^,^^18^^,^^19^ Recent analyses further suggest that some MS-associated peripheral methylation signatures are primarily attributable to B-cell and monocyte compartments,^15^^,^^18^ whereas studies of MS-discordant monozygotic twins have identified altered methylation at protocadherin loci, including PCDHγ-related regions.^87^ In contrast, our CSF-cell data point towards T-cell regulatory elements and adhesion/migration pathways in a CNS-proximal immune compartment. Thus, peripheral blood studies provide important systemic context, while the present CSF methylome highlights compartmentalised immune regulatory changes that may be more directly linked to neuroinflammation.

Despite the strengths of our comprehensive CSF methylome profiling and multi-cohort validation, several considerations remain. The sample sizes were constrained by the availability of eligible CSF specimens and the technical feasibility of generating sequencing libraries of sufficient complexity. Consequently, the small discovery cohort and lower post-deduplication CpG coverage in the validation cohort may have limited statistical power, particularly for detecting modest effects. Non-significant findings, especially in the validation cohort, should therefore not be interpreted as evidence for the absence of a biological association. Additional sequencing of low-complexity libraries was unlikely to substantially improve effective CpG coverage because it predominantly generated duplicated reads. Validation across more diverse populations will be essential to confirm the robustness and generalisability of the identified methylation signatures. Although most participants were untreated at the time of sampling, a small number received different treatments. Given the limited sample size and heterogeneous treatment types, treatment status was not included as a covariate, and potential treatment effects, as well as effects of individual genetic variation and external exposures, cannot be fully excluded. Age and sex were included as covariates in the regression models, but other unavailable variables could not be directly assessed. In addition, although our analyses implicate coordinated *PCDH* silencing by DNA methylation and integration with γ-secretase and AHR signalling, dedicated experimental studies will be required to establish causality and elucidate the underlying mechanisms. Moreover, our analyses were performed on bulk CSF immune populations, which precludes precise attribution of these alterations to specific immune subsets; future single-cell epigenomic and transcriptomic profiling will therefore be important to resolve cellular heterogeneity and define subset-specific contributions. Longitudinal studies, ideally spanning relapse–remission transitions and therapeutic intervention, may further clarify whether these methylation signatures reflect dynamic disease activity or treatment response.

In summary, this study provides a comprehensive genome-wide characterisation of the DNA methylation landscape of CSF immune cells from pwMS, revealing robust and biologically meaningful epigenetic alterations that converge on pathways regulating immune activation, cell migration, and transcriptional control. Our findings highlight enhanced migratory and adhesive programming of CSF immune cells and uncover a previously unknown role of protocadherins and their integration with γ-secretase and AHR signalling, suggesting a mechanistic axis through which environmental, metabolic, and viral cues may shape immune dysregulation in MS. These results not only advance our understanding of MS pathogenesis but also nominate candidate pathways, regulatory networks, and epigenetic signatures with potential relevance for biomarker development, precision patient stratification, and therapeutic targeting.

## Contributors

Conceptualisation: M.J., M.N., Y.H.; Data curation: Y.H., M.N., N.H., V.B.; Formal analysis: Y.H., G.Y.Z., M.N., H.L., N.H., V.B.; Funding acquisition: M.J., T.O., F.P., G.K.; Investigation: Y.H., G.Y.Z., M.N., L.K., M.J.; Methodology: Y.H., G.Y.Z., H.L., C.S., M.P.K., N.R., C.R.P., E.I.; Project administration: M.J., Mo.K., M.N.; Resources: M.J., T.O., F.P., G.K.; Supervision: M.J., M.N., G.K., Mi.K.; Visualisation: Y.H.; Writing—original draft: Y.H., M.N., L.K., M.J.; Writing—review & editing: All authors. Y.H. and M.N. had access to and verified the underlying data. All the authors read and approved the final version of the manuscript.

## Data sharing statement

The sequencing data generated in this study have been deposited in the European Genome-phenome Archive (EGA) under dataset accession number EGAD50000002813.

## Declaration of interests

F.P and T.O. report unrestricted grants for scientific research from Merck and Sanofi; F.P. reports additional unrestricted grants from Denka, Novartis, Pfizer and UCB; and T.O. has received fees or honoraria for lectures or educational events from Merck and Sanofi. F.P. and M.J. report research grants from European Union's Horizon Europe and Erling Persson's Foundation, unrelated to the current study; M.J. reports additional research grant from the German Research Foundation. F.P. is serving on a Data Safety Monitoring Board or Advisory Board for Lundbeck and Roche; and holding an unpaid role as Chairman of the Research Committee Neuro for a patient organisation. M.J. receives honoraria for serving as a Scientific Committee Member for the Swedish Brain Foundation, and holds an unpaid role as a Scientific Board Member of the European School of Neuroimmunology. All other authors declare no competing interests.

## Appendix A Supplementary data

Supplementary Figures Supplementary Fig. 1. **Overview of samples and sequencing depth**. (**A)** Total number of CpG sites covered per sample at 10× sequencing depth. **(B)** Number of retained CpG sites across samples under different minimum coverage thresholds. **(C)** Number of retained CpG sites as a function of both minimum CpG coverage and minimum sample inclusion thresholds. Supplementary Fig. 2. **Comparison of differential methylation analysis methods**. (**A)** Enrichment analysis of the top 1000 DMPs identified by Limma (first column), methylKit (second column), RADmeth (third column), and the final DMP set (fourth column), evaluated across ranked *P* values from Limma (first row), methylKit (second row), and RADmeth (third row). **(B)** Overlap of DMP candidates detected by Limma, methylKit, and RADmeth (*P* < 0.001; mean absolute methylation difference ≥0.1). **(C)** Distribution of DMR lengths.  **(D)** Overlap and correlation between DMPs and DMRs.  **(E)** Correlation of methylation levels between DMPs and DMRs. DMP: differentially methylated position, DMR: differentially methylated region. Supplementary Fig. 3. **Coverage of shallow-sequenced samples and replication of DMPs**. (**A)** Heatmap showing the number of retained CpG sites across samples at varying coverage thresholds. **(B)** Line plot illustrating CpG site retention across ten sample groups at increasing minimal coverage thresholds. **(C)** Visualisation of the DMR, MS-risk SNPs, and IHEC chromatin-state annotations at the CD37 locus. Chromatin state colours follow the standard ChromHMM 18-state annotation. **(D)** Quadrant distribution of CpG methylation differences between shallow and deep sequencing. Axes indicate methylation differences between datasets, and the shallow WGBS were filtered by an absolute methylation difference threshold of 0.05. Numbers indicate counts and percentages of CpGs in each quadrant. Supplementary Fig. 4. **Characteristics and functional annotation of differentially methylated positions (DMPs) and regions (DMRs)**. **(A)** Proportional distribution of DMPs across CpG features and genomic annotations (left), and methylation levels of DMPs within these features in RRMS and NINDC samples (right). *P* values were calculated using the two-sided Wilcoxon rank-sum test. **(B)** Proportional distribution (top) and methylation levels (bottom) of DMRs across CpG features. *P* values were calculated using two-sided Wilcoxon rank-sum tests and adjusted across CpG features using the Benjamini–Hochberg method. The central line in (A-B) indicates the median, and the upper and lower lines represent the 75th and 25th percentiles, respectively. **(C)** Proportional distribution of background CpG sites across CpG features (top) and *genomic* annotations (bottom). **(D)** GeneSetCluster analysis of pathways enriched among genes associated with DMRs (adjusted *P* < 0.05 and gene count ≥10). Heatmap shows similarity among enriched pathways with hierarchical clustering. ∗∗∗∗: BH-adjusted *P* < 0.0001, DMP: differentially methylated position, DMR: differentially methylated region. Supplementary Fig. 5. **Overlap between DNA methylation and gene expression changes**. **(A)** Scatter plots showing DNA methylation differences plotted against gene expression fold changes (log2) in RNA-sequencing and Affymetrix datasets. Analyses were performed separately for promoter-associated and all gene-associated DMPs and DMRs. Points are coloured by quadrant, representing concordant or discordant directions of methylation and expression changes; numbers and percentages indicate the proportion of features in each quadrant. **(B)** Overlap between differentially methylated genes and DEGs. **(C)** Overlap between differentially methylated genes and DEGs within inflammation-related pathways. DMP: differentially methylated position, DMR: differentially methylated region. Supplementary Fig. 6. Enrichment of DMPs and DMRs across distinct IHEC chromatin states. **(A)** Enrichment of DMPs and DMRs across 108 IHEC chromatin states. Colour intensity represents enrichment significance −log10(*P*adj). **(B)** Enrichment of DMPs and DMRs across all available IHEC T cell samples, with chromatin states shown on rows and samples on columns. **(C)** Enrichment of DMPs across IHEC T cell samples, showing a representative subset of naïve CD4^+^ T cell samples for Visualisation clarity. **(D)** Enrichment of DMPs and DMRs across ten representative IHEC T cell samples selected based on the highest variability in chromatin-state enrichment across samples, together with Th17 and Treg samples. Annotation bars indicate project, tissue type, and cell type for (B-D). Colour intensity represents enrichment significance, −log10(Padj). DMP: differentially methylated position, DMR: differentially methylated region, DM: DMPs plus DMRs, 1_TssA: Active transcription start site, 2_TssFlnk: Flanking transcription start site, 3_TssFlnkU: Upstream flanking transcription start site, 4_TssFlnkD: Downstream flanking transcription start site, 5_Tx: Strong transcription, 6_TxWk: Weak transcription, 7_EnhG1: Genic enhancer 1, 8_EnhG2: Genic enhancer 2, 9_EnhA1: Active enhancer 1, 10_EnhA2: Active enhancer 2, 11_EnhWk: Weak enhancer, 12_ZNF/Rpts: ZNF genes and repeats, 13_Het: Heterochromatin, 14_TssBiv: Bivalent/poised transcription start site, 15_EnhBiv: Bivalent enhancer, 16_ReprPC: Polycomb-repressed region, 17_ReprPCWk: Weak Polycomb repression, 18_Quies: Quiescent/low signal region, DP thymocytes: Double-positive thymocytes, CD4 thymocyte: CD4^+^ thymocytes, CD8 thymocyte: CD8^+^ thymocytes, Naive CD4: Naive CD4^+^ T cells, Naive CD8: Naive CD8^+^ T cells, Treg: Regulatory T cells, Th17: T helper 17 cells, CD4CM: CD4^+^ central memory T cells, CD4EM: CD4^+^ effector memory T cells, CD4TEM: CD4^+^ effector memory T cells re-expressing CD45RA, CD4 memory: CD4^+^ memory T cells, CD8CM: CD8^+^ central memory T cells, CD8EM: CD8^+^ effector memory T cells, CD8TEM: CD8^+^ effector memory T cells re-expressing CD45RA, CD8 memory: CD8^+^ memory T cells. Supplementary Fig. 7. **Gene overlaps across ten T cell samples within four IHEC genomic features**. UpSet plots showing overlaps of genes associated with differential methylation across ten T cell samples within four IHEC genomic features: TSS, enhancer (Enh), bivalent enhancer (EnhBiv), and repressive Polycomb (ReprPC). Bar plots indicate the size of gene intersections, while the dot matrix denotes the gene numbers of T cell samples contributing to each intersection. Supplementary Fig. 8. **Flow cytometric analysis of PCDHγ expression, co-expression network analysis, and γ-secretase gene expression across T cell subtypes**. **(A)** Representative gating strategy for flow cytometric analysis of pan-protocadherin gamma (PCDHγ) expression. Cells were sequentially gated on lymphocytes (forward scatter area, FSC-A, versus side scatter area, SSC-A), single cells, live cells, and CD3^+^ T cells, followed by analysis of pan-PCDHγ expression plotted against CD20. **(B)** Flow cytometric analysis of pan-PCDHγ staining under surface-only conditions and after surface staining followed by secondary antibody incubation in CD3^+^ T cells from two donors. Gates indicate pan-PCDHγ^+^ cells, and percentages represent the proportion of pan-PCDHγ^+^ cells among CD3^+^ T cells. **(C)** Enriched pathways of PCDH co-expressed genes. Pairwise gene correlations were calculated using Pearson correlation, and significantly co-expressed gene pairs (Padj <0.001) were used for pathway enrichment analysis. **(D)** Boxplots showing RNA expression levels of γ-secretase complex genes (APH1A, APH1B, NCSTN, PSEN1, PSEN2, and PSENEN) across T cell subtypes. Boxplots show the median (centre line) and interquartile range (box), whiskers extend to 1.5 × IQR, and points represent individual samples. Supplementary Fig. 9. **Expression of PCDH genes across T cell subsets**. Boxplots showing log_2_ (TPM + 1) transformed RNA-seq expression levels of expressed PCDH genes with detectable expression across ten T cell subtypes. Boxplots show the median (centre line) and interquartile range (box), whiskers extend to 1.5 × IQR, and points represent individual samples. Supplementary Fig. 10. **Flow cytometry gating strategy and TNFα expression in CD4^+^ T-cell subsets**. (**A**) Representative gating strategy for identifying PCDHγ^+^ cells within CD4^+^ T-cell subsets. Single cells were first gated by FSC-A/FSC-H, followed by live cells, CD3^+^ T cells, and CD4^+^ T cells. CD4^+^ T cells were further separated into CD45RO^+^ and CD45RO^−^ populations, and PCDHγ expression was assessed in each subset. (**B**) Representative flow cytometry plots showing TNFα expression in CD45RO^+^ CD4^+^ T cells under control, PMA/ionomycin, and anti-CD3 stimulation conditions. (**C**) Representative flow cytometry plots showing TNFα expression in CD45RO^−^ CD4^+^ T cells under the same stimulation conditions. Numbers indicate the percentage of cells within the indicated gate.
