# Supplementary material for: DNA methylation landscape of cerebrospinal fluid cells in multiple sclerosis: an epigenome-wide association study
Source: eBioMedicine. 2026 Aug 27;131:106454. doi: 10.1016/j.ebiom.2026.106454 (PMC13545696; doi:10.1016/j.ebiom.2026.106454)

Sup. Figure 1

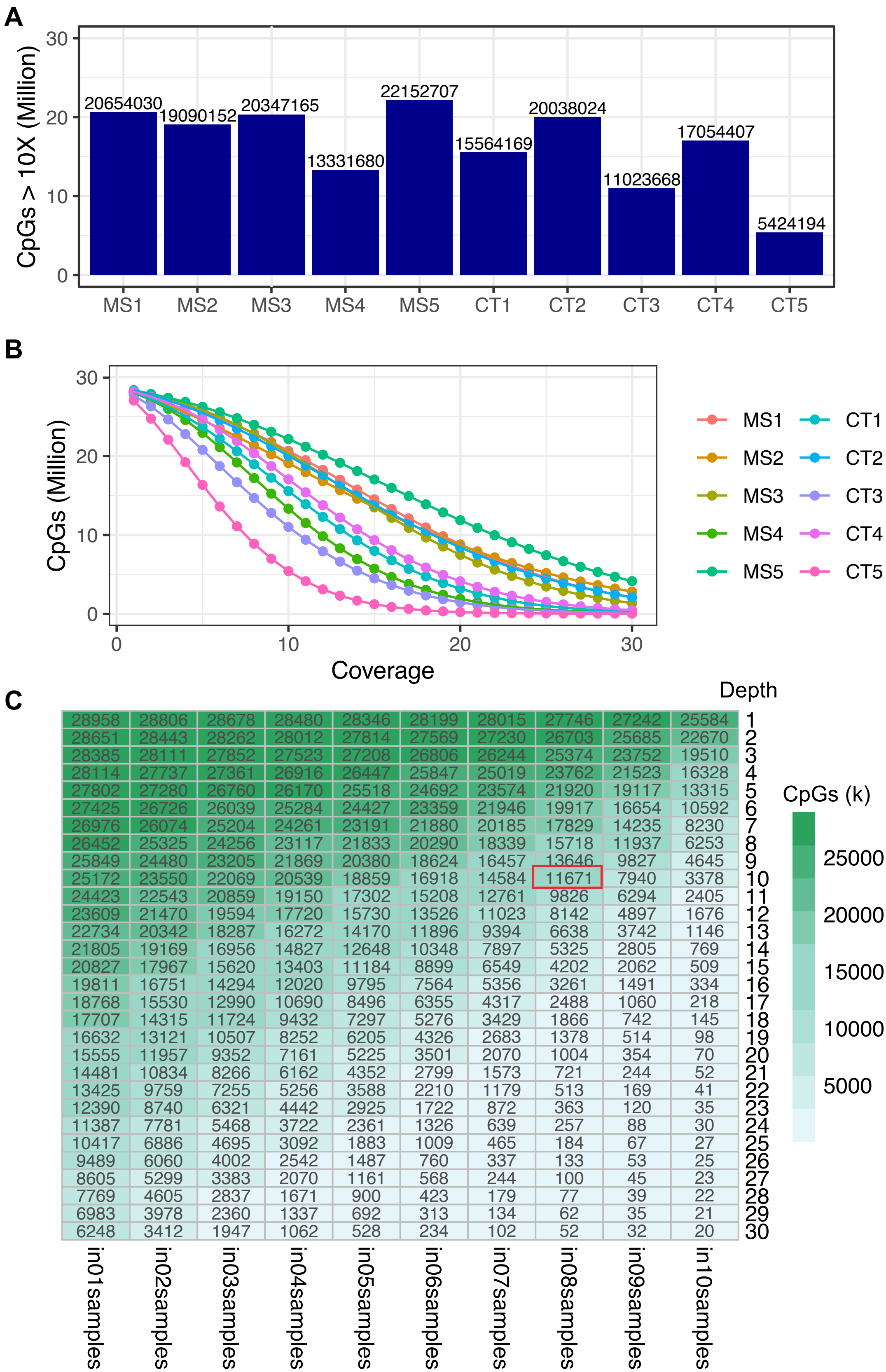
A
CpGs > 10X (Million)
0
10
20
30
20654030
19090152
20347165
13331680
22152707
15564169
20038024
11023668
17054407
5424194
MS1
MS2
MS3
MS4
MS5
CT1
CT2
CT3
CT4
CT5
B
CpGs (Million)
Coverage
MS1
MS2
MS3
MS4
MS5
CT1
CT2
CT3
CT4
CT5
C
Depth
CpGs (k)
25000
20000
15000
10000
5000
1 28958 28806 28678 28480 28346 28199 28015 27746 27242 25584
2 28651 28443 28262 28012 27814 27569 27230 26703 25685 22670
3 28385 28111 27852 27523 27208 26806 26244 25374 23752 19510
4 28114 27737 27361 26916 26447 25847 25019 23762 21523 16328
5 27802 27280 26760 26170 25518 24692 23574 21920 19117 13315
6 27425 26726 26039 25284 24427 23359 21946 19917 16654 10592
7 26976 26074 25204 24261 23191 21880 20185 17829 14235 8230
8 26452 25325 24256 23117 21833 20290 18339 15718 11937 6253
9 25849 24480 23205 21869 20380 18624 16457 13646 9827 4645
10 25172 23550 22069 20539 18859 16918 14584 11671 7940 3378
11 24423 22543 20859 19150 17302 15208 12761 9826 6294 2405
12 23609 21470 19594 17720 15730 13526 11023 8142 4897 1676
13 22734 20342 18287 16272 14170 11896 9394 6638 3742 1146
14 21805 19169 16956 14827 12648 10348 7897 5325 2805 769
15 20827 17967 15620 13403 11184 8899 6549 4202 2062 509
16 19811 16751 14294 12020 9795 7564 5356 3261 1491 334
17 18768 15530 12990 10690 8496 6355 4317 2488 1060 218
18 17707 14315 11724 9432 7297 5276 3429 1866 742 145
19 16632 13121 10507 8252 6205 4326 2683 1378 514 98
20 15555 11957 9352 7161 5225 3501 2070 1004 354 70
21 14481 10834 8266 6162 4352 2799 1573 721 244 52
22 13425 9759 7255 5256 3588 2210 1179 513 169 41
23 12390 8740 6321 4442 2925 1722 872 363 120 35
24 11387 7781 5468 3722 2361 1326 639 257 88 30
25 10417 6886 4695 3092 1883 1009 465 184 67 27
26 9489 6060 4002 2542 1487 760 337 133 53 25
27 8605 5299 3383 2070 1161 568 244 100 45 23
28 7769 4605 2837 1671 900 423 179 77 39 22
29 6983 3978 2360 1337 692 313 134 62 35 21
30 6248 3412 1947 1062 528 234 102 52 32 20
in01samples
in02samples
in03samples
in04samples
in05samples
in06samples
in07samples
in08samples
in09samples
in10samples

Sup. Figure 2

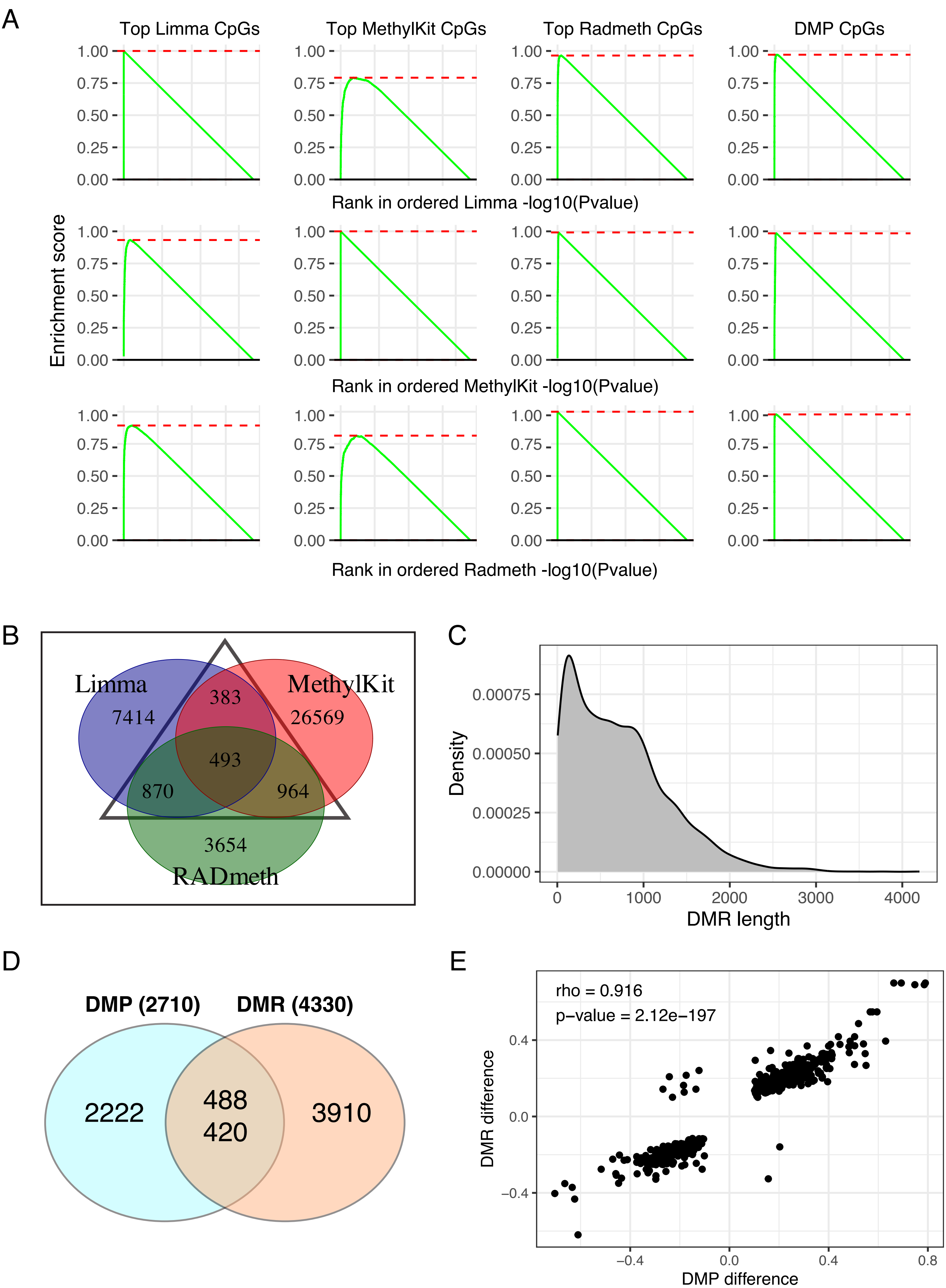
A
Top Limma CpGs
Top MethylKit CpGs
Top Radmeth CpGs
DMP CpGs
Enrichment score
1.00
0.75
0.50
0.25
0.00
Rank in ordered Limma -log10(Pvalue)
Rank in ordered MethylKit -log10(Pvalue)
Rank in ordered Radmeth -log10(Pvalue)
B
Limma
7414
MethylKit
26569
383
493
870
964
3654
RADmeth
C
Density
0.00075
0.00050
0.00025
0.00000
0
1000
2000
3000
4000
DMR length
D
DMP (2710)
DMR (4330)
2222
488
420
3910
E
rho = 0.916
p-value = 2.12e−197
DMR difference
0.4
0.0
−0.4
DMP difference
−0.4
0.0
0.4
0.8

Sup. Figure 3

A

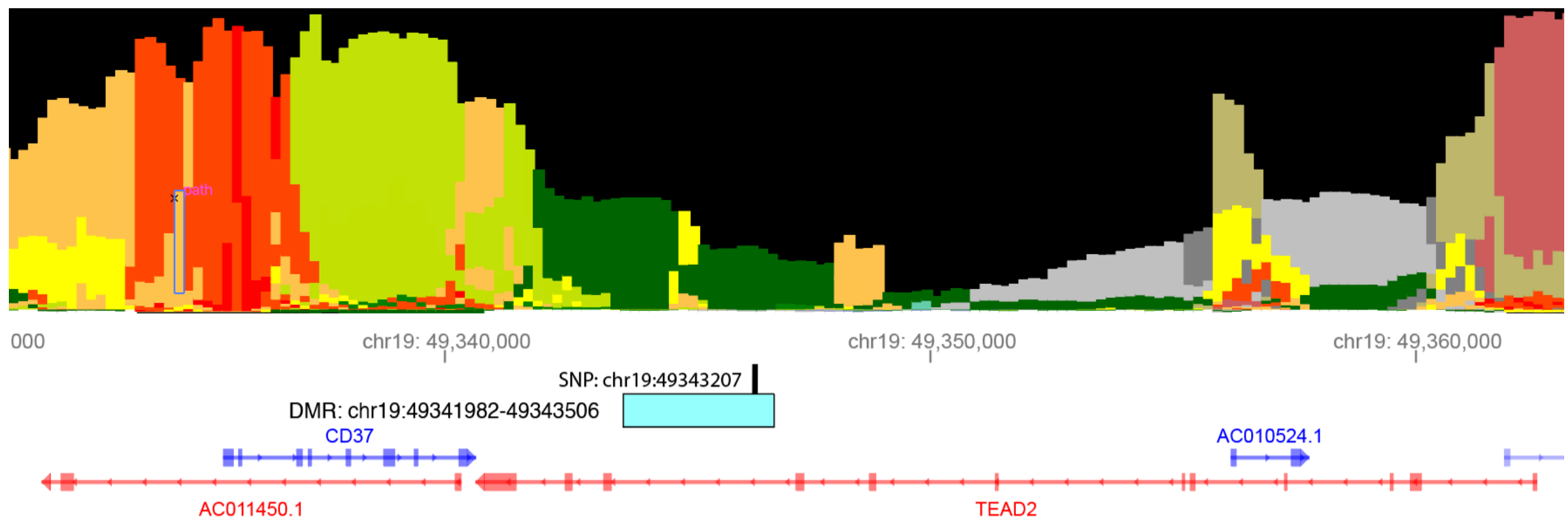

B

	in01samples	in02samples	in03samples	in04samples	in05samples	in06samples	in07samples	in08samples	in09samples	in10samples
cov01	25861120	24956905	23909712	22613979	20989840	18936276	16328785	13006597	8853722	4051888
cov02	22956086	20163931	17419669	14632520	11800841	8975647	6253333	3783971	1793150	510578
cov03	18347722	13749838	10129669	7163835	4762100	2899476	1557010	695659	235357	51291
cov04	13044654	7820045	4651485	2631486	1375473	645760	265247	94027	30564	10746
cov05	8258135	3702255	1683595	728849	294758	111463	42495	18952	10431	5957
cov06	4683922	1471622	489216	163712	58116	24942	14095	9540	6688	4184
cov07	2410597	500636	125562	39767	18508	11971	8858	6702	4917	3159
cov08	1144132	156642	36778	16495	11023	8304	6474	5022	3774	2505
cov09	512179	51957	16651	10635	8052	6290	5025	3914	3004	2026
cov10	222905	22327	10981	7984	6175	4982	3996	3169	2428	1669
cov11	97375	13329	8318	6318	4982	4026	3233	2622	1997	1423
cov12	45517	9712	6677	5088	4046	3248	2672	2176	1706	1253
cov13	24222	7715	5466	4178	3343	2693	2253	1864	1484	1103
cov14	14951	6267	4476	3450	2832	2324	1956	1617	1319	949
cov15	10592	5201	3742	2973	2457	2050	1728	1417	1159	861
cov16	8323	4437	3252	2608	2173	1808	1514	1255	1052	791
cov17	6789	3794	2826	2285	1913	1606	1367	1163	951	714
cov18	5722	3314	2493	2024	1713	1448	1235	1051	863	647
cov19	4899	2853	2219	1791	1531	1297	1126	946	788	583
cov20	4222	2521	1980	1606	1366	1178	1031	878	727	529

Color scale: 2.5e+07, 2e+07, 1.5e+07, 1e+07, 5e+06

C

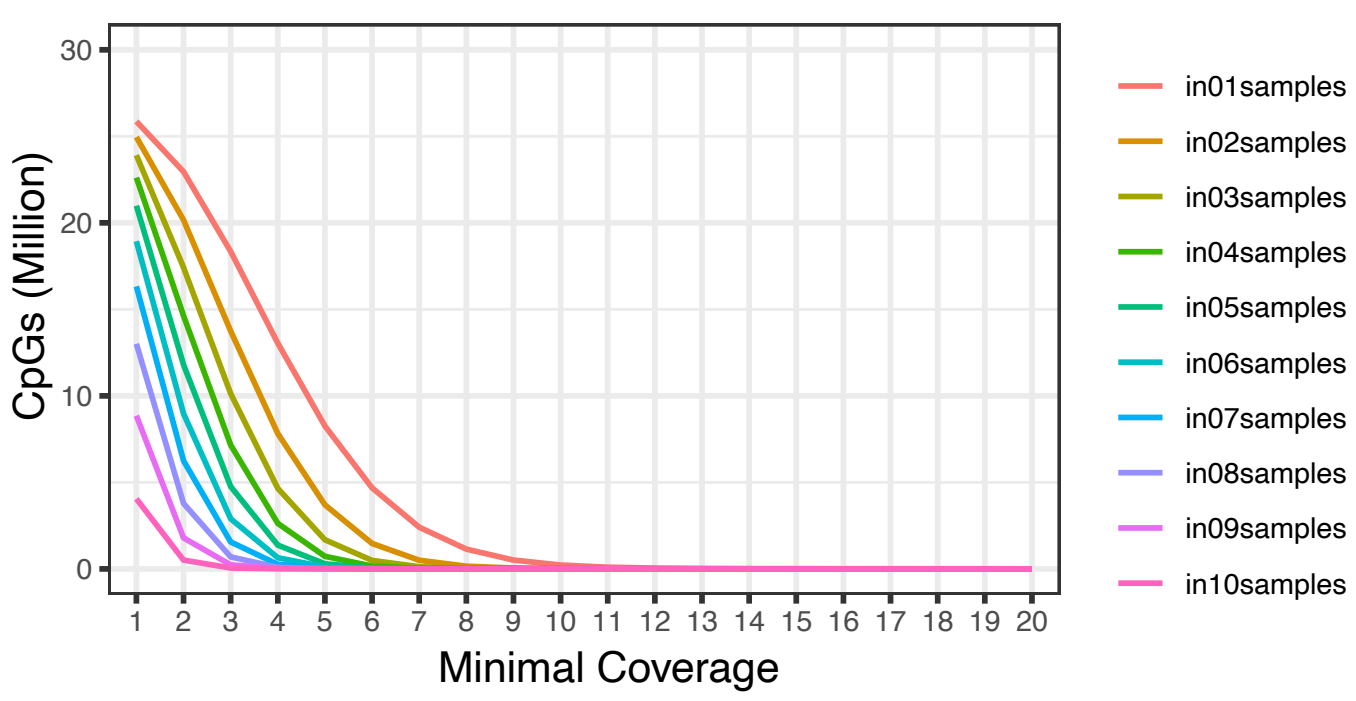

D

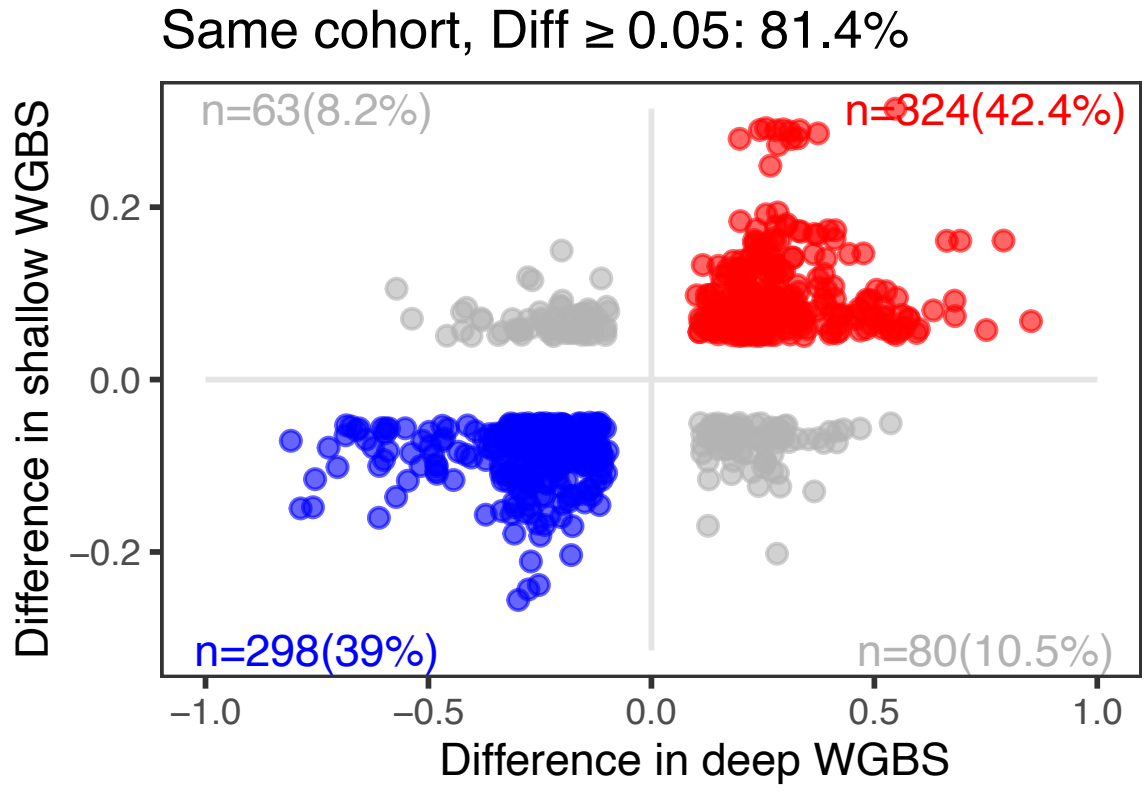

Sup. Figure 4

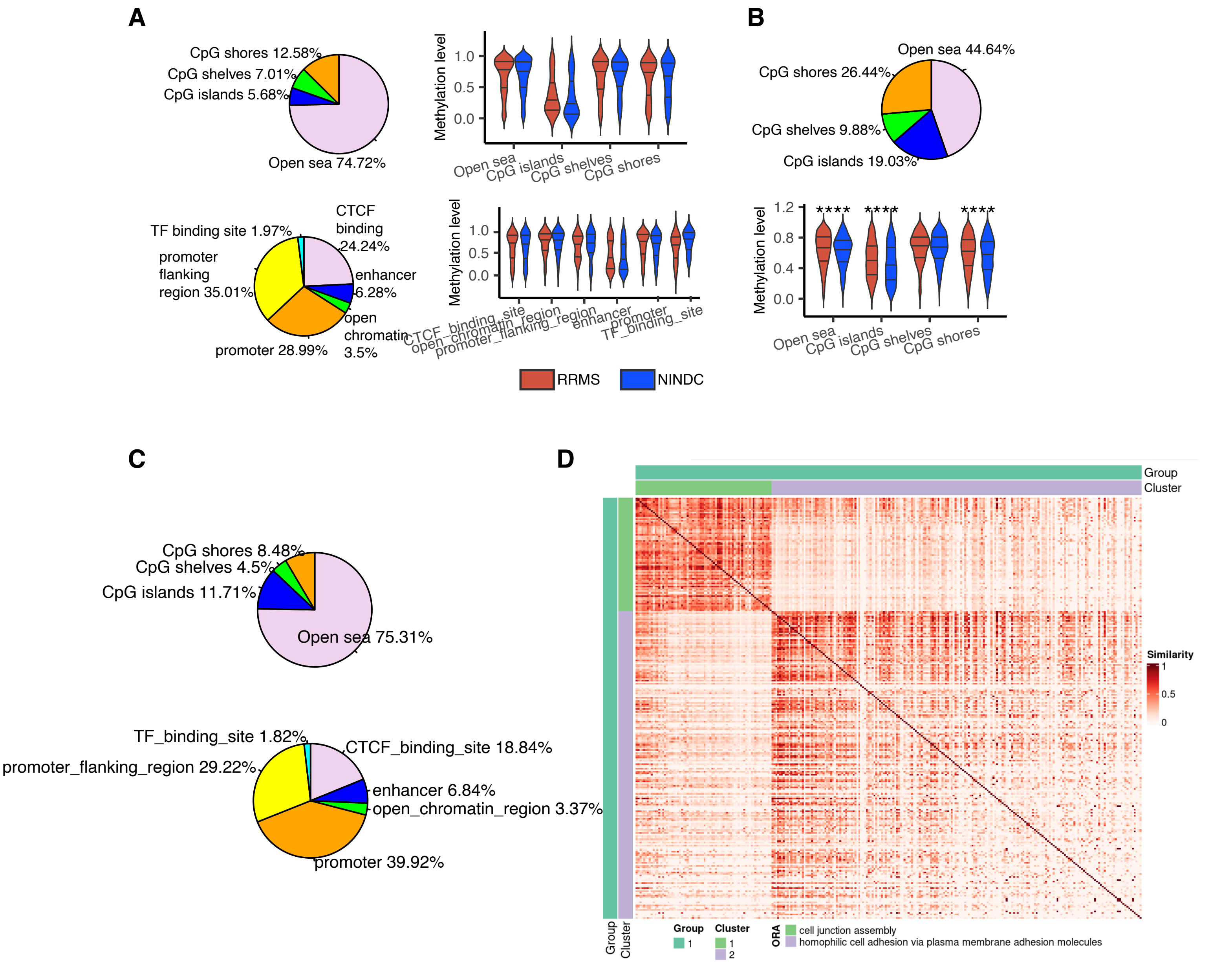

A
CpG shores 12.58%
CpG shelves 7.01%
CpG islands 5.68%
Open sea 74.72%
Methylation level
1.0
0.5
0.0
Open sea
CpG islands
CpG shelves
CpG shores
TF binding site 1.97%
CTCF binding 24.24%
promoter flanking region 35.01%
enhancer 6.28%
open chromatin 3.5%
promoter 28.99%
CTCF_binding_site
open_chromatin_region
promoter_flanking_region
enhancer
promoter
TF_binding_site
RRMS
NINDC
B
Open sea 44.64%
CpG shores 26.44%
CpG shelves 9.88%
CpG islands 19.03%
1.2
0.8
0.4
0.0
****
****
****
C
CpG shores 8.48%
CpG shelves 4.5%
CpG islands 11.71%
Open sea 75.31%
TF_binding_site 1.82%
CTCF_binding_site 18.84%
promoter_flanking_region 29.22%
enhancer 6.84%
open_chromatin_region 3.37%
promoter 39.92%
D
Group
Cluster
Similarity
1
0.5
0
ORA
cell junction assembly
homophilic cell adhesion via plasma membrane adhesion molecules
2

Sup. Figure 5

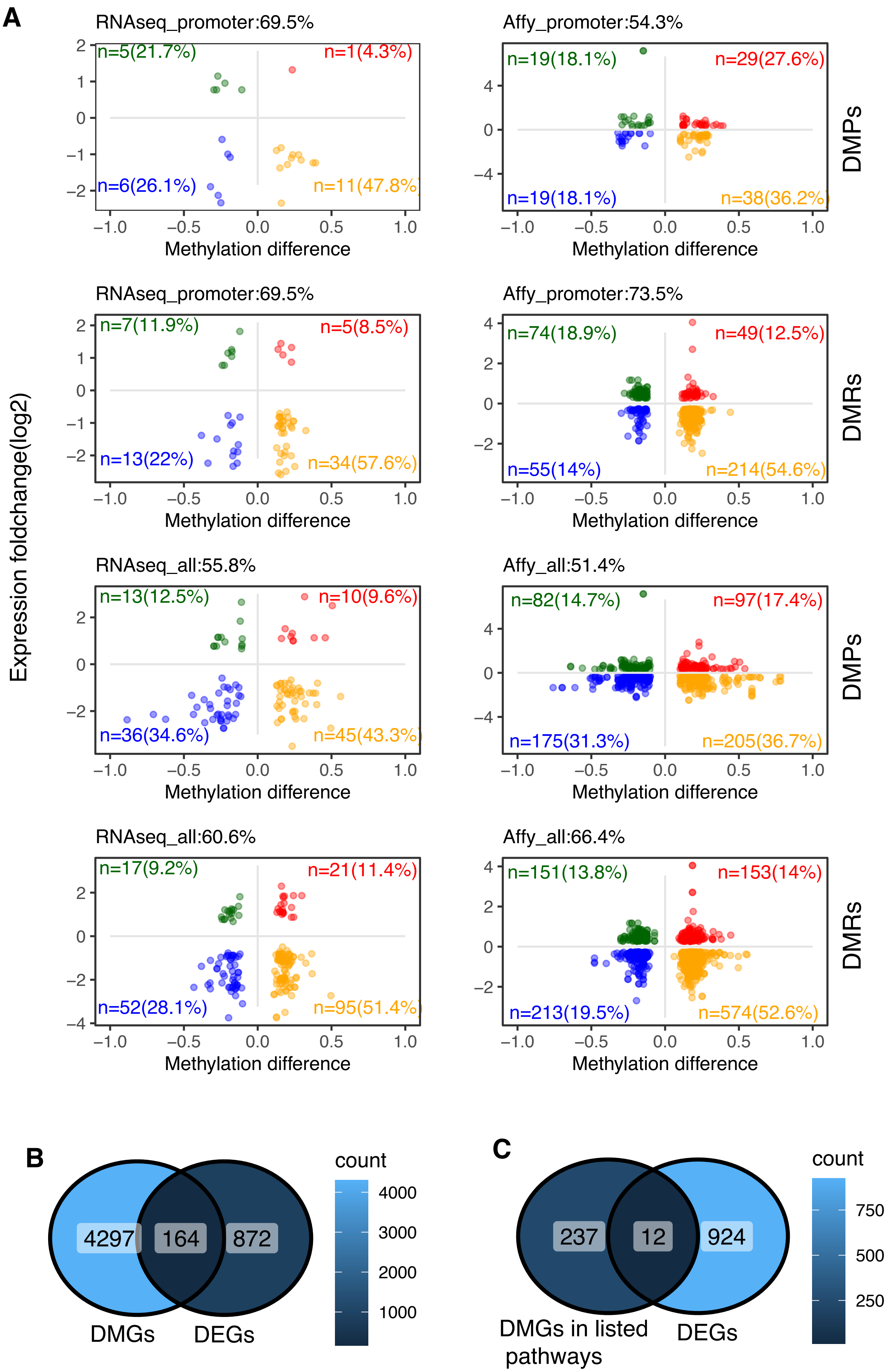

A
RNAseq_promoter:69.5%
n=5(21.7%)
n=1(4.3%)
n=6(26.1%)
n=11(47.8%)
Affy_promoter:54.3%
n=19(18.1%)
n=29(27.6%)
n=19(18.1%)
n=38(36.2%)
DMPs
RNAseq_promoter:69.5%
n=7(11.9%)
n=5(8.5%)
n=13(22%)
n=34(57.6%)
Affy_promoter:73.5%
n=74(18.9%)
n=49(12.5%)
n=55(14%)
n=214(54.6%)
DMRs
RNAseq_all:55.8%
n=13(12.5%)
n=10(9.6%)
n=36(34.6%)
n=45(43.3%)
Affy_all:51.4%
n=82(14.7%)
n=97(17.4%)
n=175(31.3%)
n=205(36.7%)
DMPs
RNAseq_all:60.6%
n=17(9.2%)
n=21(11.4%)
n=52(28.1%)
n=95(51.4%)
Affy_all:66.4%
n=151(13.8%)
n=153(14%)
n=213(19.5%)
n=574(52.6%)
DMRs
Expression foldchange(log2)
Methylation difference
B
4297
164
872
DMGs
DEGs
count
4000
3000
2000
1000
C
237
12
924
DMGs in listed pathways
DEGs
count
750
500
250

Sup. Figure 6

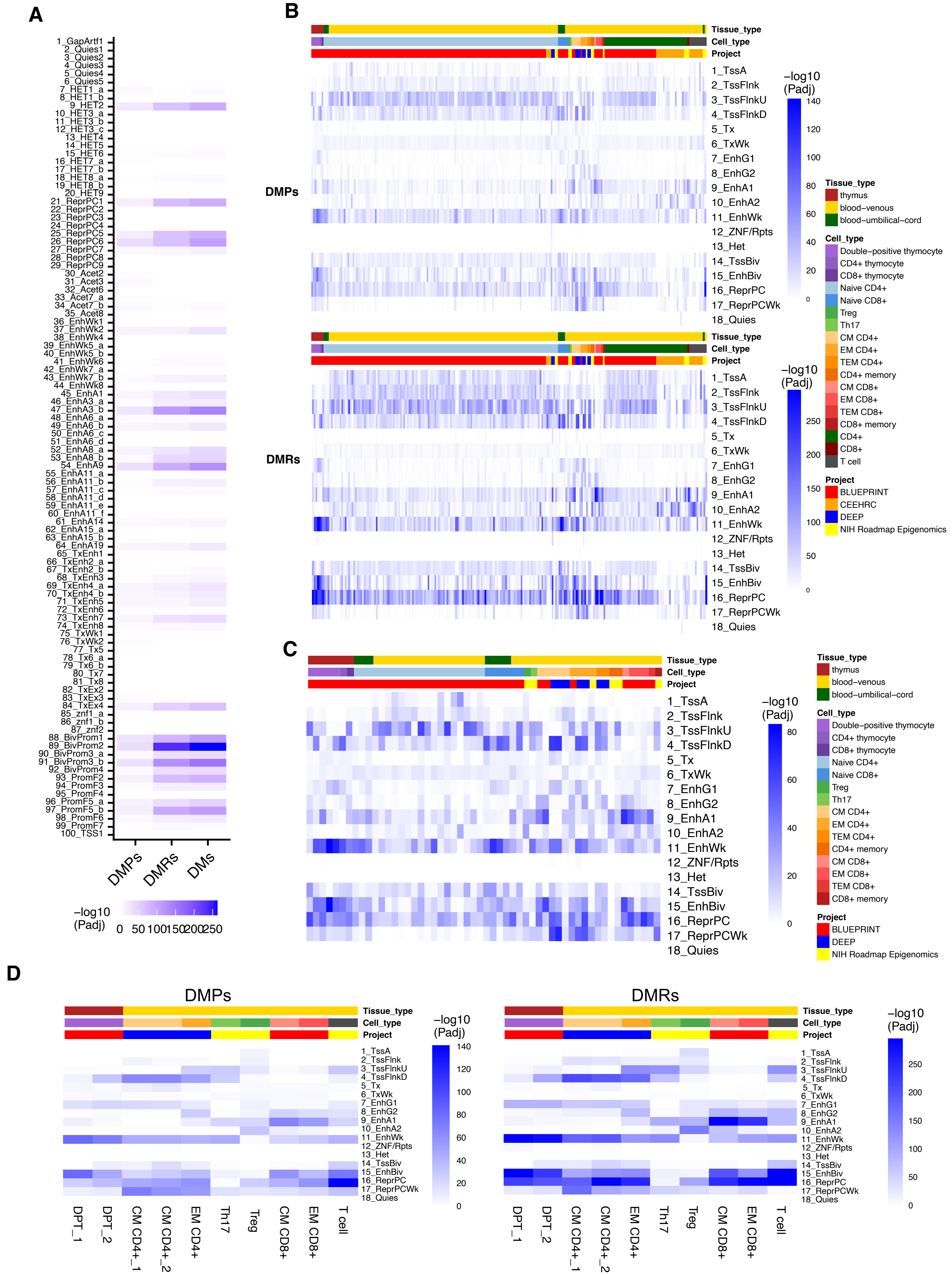
A
1_GapArtf1
2_Quies1
3_Quies2
4_Quies3
5_Quies4
6_Quies5
7_HET1_a
8_HET1_b
9_HET2
10_HET3_a
11_HET3_b
12_HET3_c
13_HET4
14_HET5
15_HET6
16_HET7_a
17_HET7_b
18_HET8_a
19_HET8_b
20_HET9
21_ReprPC1
22_ReprPC2
23_ReprPC3
24_ReprPC4
25_ReprPC5
26_ReprPC6
27_ReprPC7
28_ReprPC8
29_ReprPC9
30_Acet2
31_Acet3
32_Acet6
33_Acet7_a
34_Acet7_b
35_Acet8
36_EnhWk1
37_EnhWk2
38_EnhWk4
39_EnhWk5_a
40_EnhWk5_b
41_EnhWk6
42_EnhWk7_a
43_EnhWk7_b
44_EnhWk8
45_EnhA1
46_EnhA3_a
47_EnhA3_b
48_EnhA6_a
49_EnhA6_b
50_EnhA6_c
51_EnhA6_d
52_EnhA8_a
53_EnhA8_b
54_EnhA9
55_EnhA11_a
56_EnhA11_b
57_EnhA11_c
58_EnhA11_d
59_EnhA11_e
60_EnhA11_f
61_EnhA14
62_EnhA15_a
63_EnhA15_b
64_EnhA19
65_TxEnh1
66_TxEnh2_a
67_TxEnh2_b
68_TxEnh3
69_TxEnh4_a
70_TxEnh4_b
71_TxEnh5
72_TxEnh6
73_TxEnh7
74_TxEnh8
75_TxWk1
76_TxWk2
77_Tx5
78_Tx6_a
79_Tx6_b
80_Tx7
81_Tx8
82_TxEx2
83_TxEx3
84_TxEx4
85_znf1_a
86_znf1_b
87_znf2
88_BivProm1
89_BivProm2
90_BivProm3_a
91_BivProm3_b
92_BivProm4
93_PromF2
94_PromF3
95_PromF4
96_PromF5_a
97_PromF5_b
98_PromF6
99_PromF7
100_TSS1
DMPs
DMRs
DMs
−log10 (Padj)
0 50 100 150 200 250
B
DMPs
DMRs
Tissue_type
Cell_type
Project
1_TssA
2_TssFlnk
3_TssFlnkU
4_TssFlnkD
5_Tx
6_TxWk
7_EnhG1
8_EnhG2
9_EnhA1
10_EnhA2
11_EnhWk
12_ZNF/Rpts
13_Het
14_TssBiv
15_EnhBiv
16_ReprPC
17_ReprPCWk
18_Quies
−log10 (Padj)
140 120 100 80 60 40 20 0
250 200 150 100 50 0
Tissue_type
thymus
blood−venous
blood−umbilical−cord
Cell_type
Double−positive thymocyte
CD4+ thymocyte
CD8+ thymocyte
Naive CD4+
Naive CD8+
Treg
Th17
CM CD4+
EM CD4+
TEM CD4+
CD4+ memory
CM CD8+
EM CD8+
TEM CD8+
CD8+ memory
CD4+
CD8+
T cell
Project
BLUEPRINT
CEEHRC
DEEP
NIH Roadmap Epigenomics
C
Tissue_type
Cell_type
Project
1_TssA
2_TssFlnk
3_TssFlnkU
4_TssFlnkD
5_Tx
6_TxWk
7_EnhG1
8_EnhG2
9_EnhA1
10_EnhA2
11_EnhWk
12_ZNF/Rpts
13_Het
14_TssBiv
15_EnhBiv
16_ReprPC
17_ReprPCWk
18_Quies
−log10 (Padj)
80 60 40 20 0
Tissue_type
thymus
blood−venous
blood−umbilical−cord
Cell_type
Double−positive thymocyte
CD4+ thymocyte
CD8+ thymocyte
Naive CD4+
Naive CD8+
Treg
Th17
CM CD4+
EM CD4+
TEM CD4+
CD4+ memory
CM CD8+
EM CD8+
TEM CD8+
CD8+ memory
Project
BLUEPRINT
DEEP
NIH Roadmap Epigenomics
D
DMPs
DMRs
Tissue_type
Cell_type
Project
1_TssA
2_TssFlnk
3_TssFlnkU
4_TssFlnkD
5_Tx
6_TxWk
7_EnhG1
8_EnhG2
9_EnhA1
10_EnhA2
11_EnhWk
12_ZNF/Rpts
13_Het
14_TssBiv
15_EnhBiv
16_ReprPC
17_ReprPCWk
18_Quies
−log10 (Padj)
140 120 100 80 60 40 20 0
250 200 150 100 50 0
DPT_1
DPT_2
CM CD4+_1
CM CD4+_2
EM CD4+
Th17
Treg
CM CD8+
EM CD8+
T cell

Sup. Figure 7

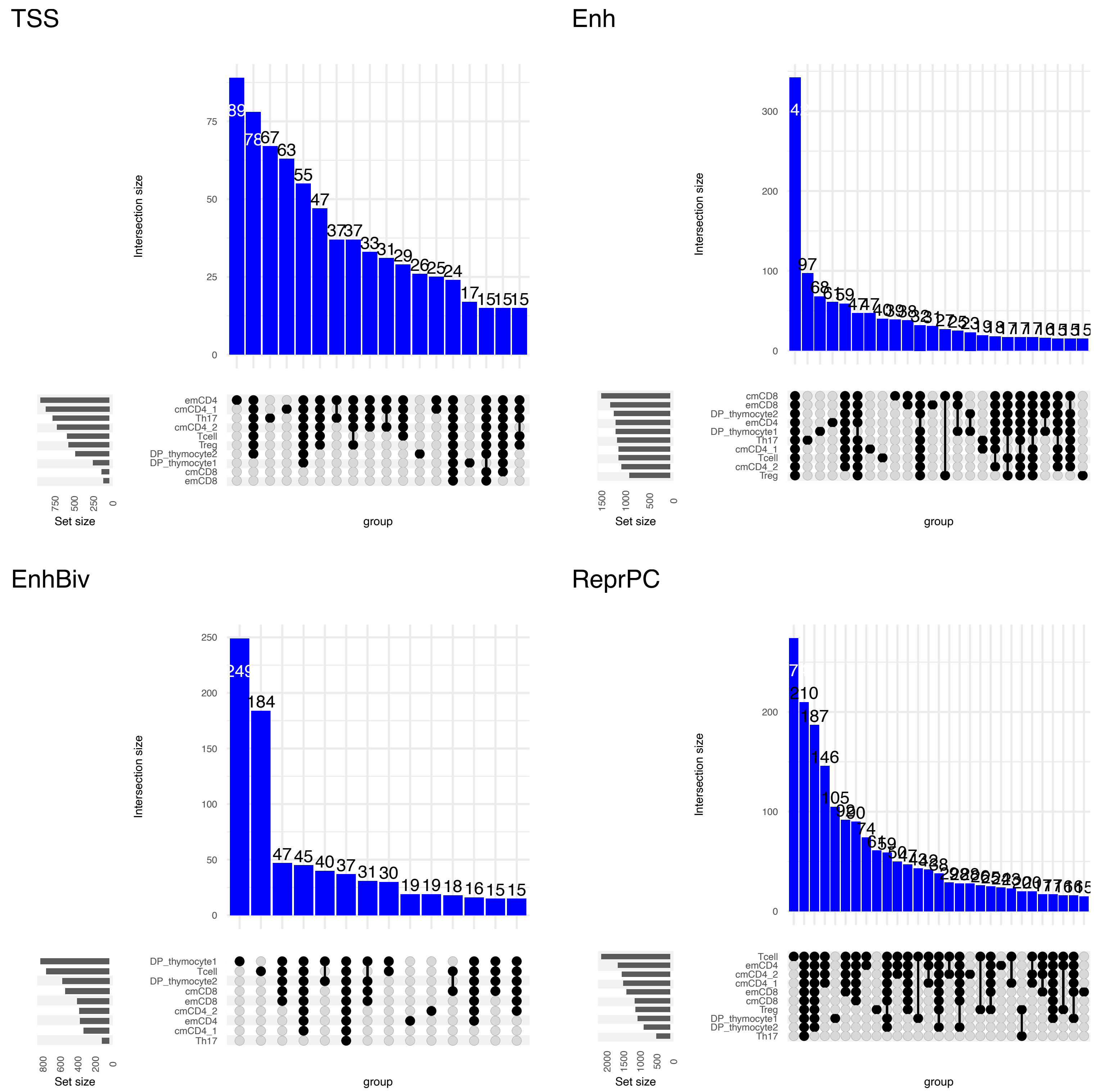
TSS
Enh
EnhBiv
ReprPC
Intersection size
Set size
group
emCD4
cmCD4_1
Th17
cmCD4_2
Tcell
Treg
DP_thymocyte2
DP_thymocyte1
cmCD8
emCD8

Sup. Figure 8

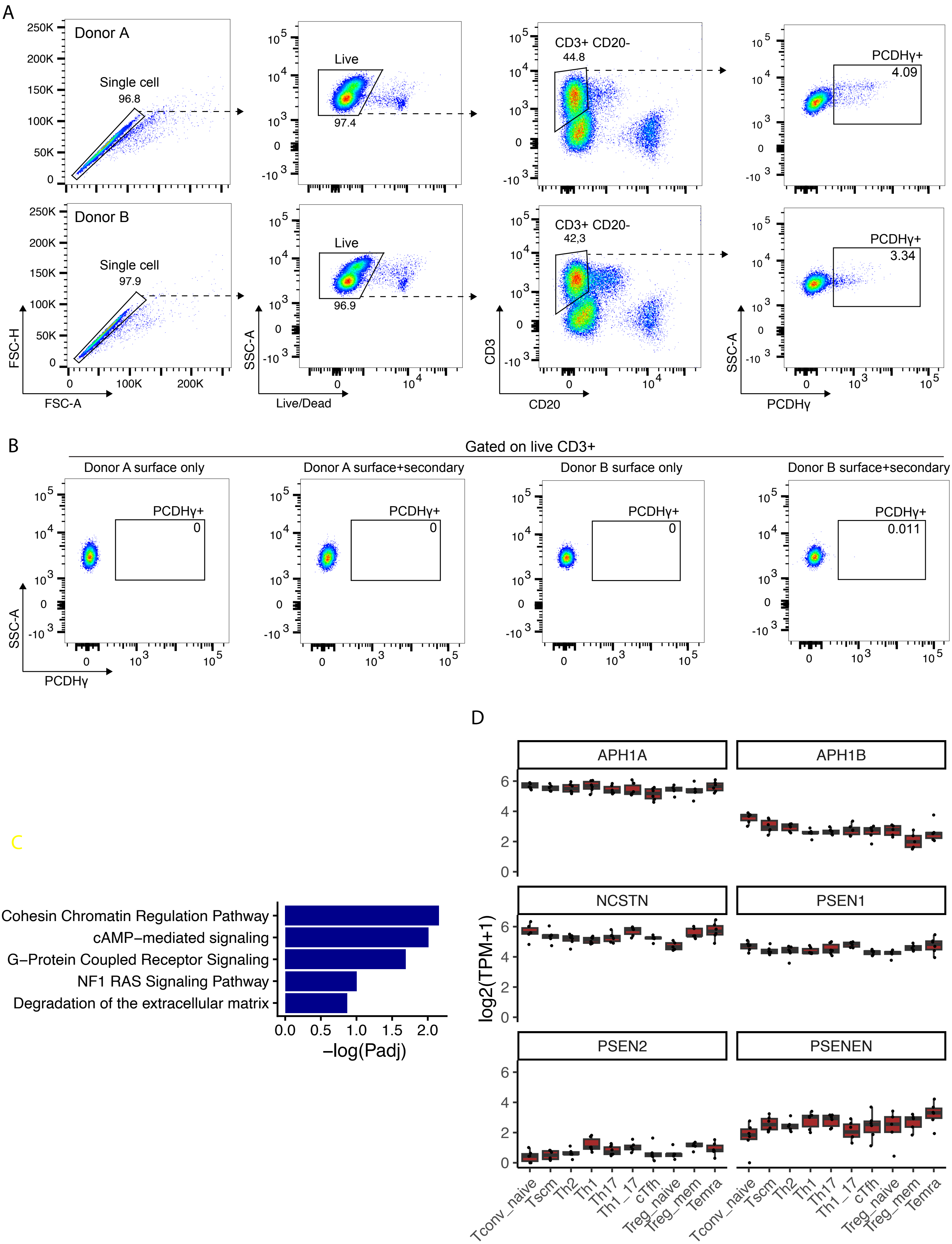

A
Donor A
Single cell
96.8
Live
97.4
CD3+ CD20-
44.8
PCDHγ+
4.09
Donor B
Single cell
97.9
Live
96.9
CD3+ CD20-
42,3
PCDHγ+
3.34
FSC-H
FSC-A
SSC-A
Live/Dead
CD3
CD20
SSC-A
PCDHγ
B
Gated on live CD3+
Donor A surface only
Donor A surface+secondary
Donor B surface only
Donor B surface+secondary
PCDHγ+
0
0
0
0.011
SSC-A
PCDHγ
C
Cohesin Chromatin Regulation Pathway
cAMP−mediated signaling
G−Protein Coupled Receptor Signaling
NF1 RAS Signaling Pathway
Degradation of the extracellular matrix
−log(Padj)
D
APH1A
APH1B
NCSTN
PSEN1
PSEN2
PSENEN
log2(TPM+1)
Tconv_naive
Tscm
Th2
Th1
Th17
Th1_17
cTfh
Treg_naive
Treg_mem
Temra

Sup. Figure 9

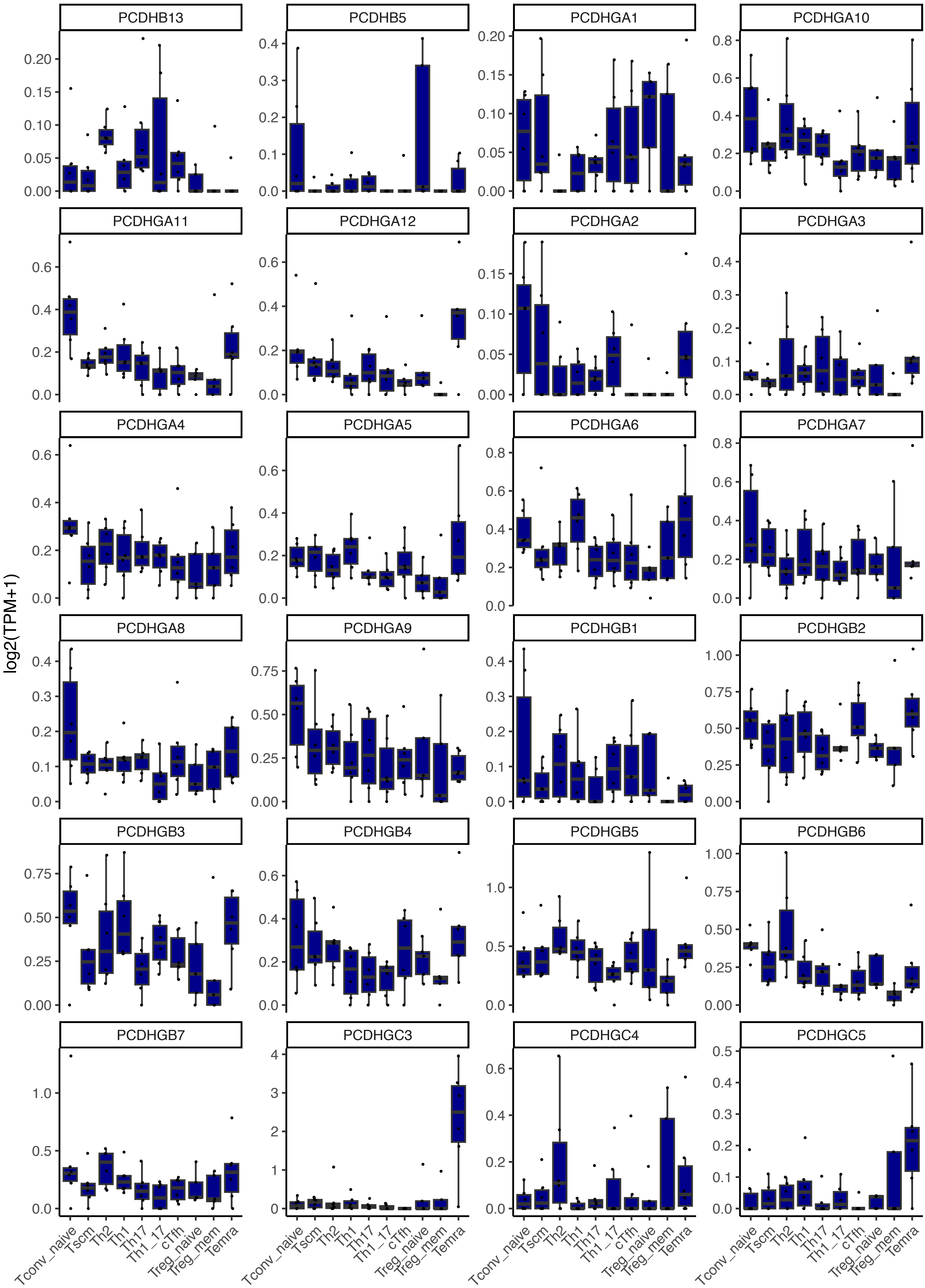
PCDHB13
PCDHB5
PCDHGA1
PCDHGA10
PCDHGA11
PCDHGA12
PCDHGA2
PCDHGA3
PCDHGA4
PCDHGA5
PCDHGA6
PCDHGA7
PCDHGA8
PCDHGA9
PCDHGB1
PCDHGB2
PCDHGB3
PCDHGB4
PCDHGB5
PCDHGB6
PCDHGB7
PCDHGC3
PCDHGC4
PCDHGC5
log2(TPM+1)
Tconv_naive
Tscm
Th2
Th1
Th17
Th1_17
cTfh
Treg_naive
Treg_mem
Temra

Sup. Figure 10

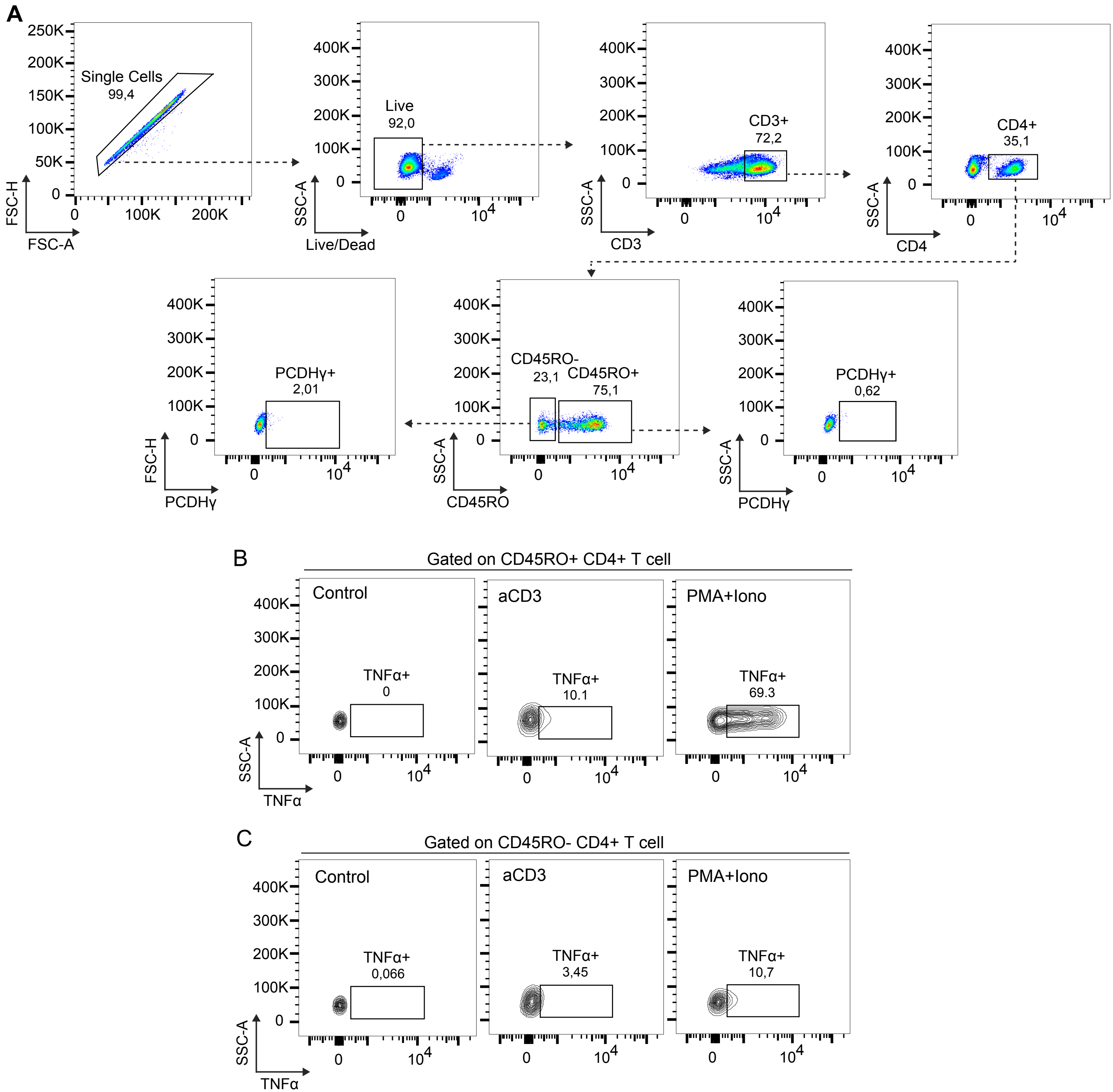

Supplement: Supplementary file 1 [file mmc1.pdf]
